# Targeted Protein Degradation: Clinical Advances in the Field of Oncology

**DOI:** 10.3390/ijms232315440

**Published:** 2022-12-06

**Authors:** Abdelrahman K. A. A. Salama, Marija V. Trkulja, Emilio Casanova, Iris Z. Uras

**Affiliations:** Department of Pharmacology, Center of Physiology and Pharmacology & Comprehensive Cancer Center (CCC), Medical University of Vienna, 1090 Vienna, Austria

**Keywords:** cancer, targeted protein degradation, degrader molecules, clinical trials

## Abstract

The field of targeted protein degradation (TPD) is a rapidly developing therapeutic modality with the promise to tame disease-relevant proteins in ways that are difficult or impossible to tackle with other strategies. While we move into the third decade of TPD, multiple degrader drugs have entered the stage of the clinic and many more are expected to follow. In this review, we provide an update on the most recent advances in the field of targeted degradation with insights into possible clinical implications for cancer prevention and treatment.

## 1. Introduction

Cancer is a major public health problem. Each year nearly 20 million new cases are diagnosed and almost 10 million deaths from cancer are registered [1,2]. The standard of care for patients includes not only surgery and radiotherapy but also chemotherapy; poor selectivity and acquired resistance limit its clinical application. Tumors harbor different underlying genetic causes and express different proteins in each patient. This heterogeneity of cancer paves the ground for precision and personalized medicine. In the past two decades, the focus of novel drug development has thus shifted towards the identification and targeting of molecular differences/drivers between tumors. A major class of targeted therapies employs monoclonal antibodies and small molecules that act by blocking the activity of pathogenic proteins that drive tumorigenesis [3]. To date, the therapeutic applicability of monoclonal antibodies is restricted to cell surface proteins [4]. Both multi-targeted and highly selective small-molecule compounds are applied in advanced treatment-resistant tumors, many being approved for early clinical settings as adjuvant therapies or as first-line for metastatic or recurrent disease [5]. Despite their success, the response is not durable due to development of resistance (i) by increasing the abundance of the target protein; (ii) by rewiring survival pathways in the complex cell-signaling milieu; or (iii) by acquisition of new mutations within the target protein that allow the malignant cell to escape the inhibitory effects of the compound. A broad range of targets such as transcription factors, scaffolding and regulatory proteins have remained undruggable due to the lack of suitable binding pockets that directly modulate protein function. Another limitation is presented by high drug concentrations to ensure active-site occupancy and to sustain clinical benefit in vivo, which in turn increases the risk of off-target effects [6]. One way to reduce intracellular protein concentrations is through gene-editing technologies using therapeutic nucleic acids including antisense oligonucleotides (ASO), small interfering RNA (siRNA) and CRISPR-Cas9 [6]. To date ten ASO therapeutics [7] and four siRNA-based therapies [8] have been approved by the Food and Drug Administration (FDA) for their use in different disease conditions, but none has been approved to treat cancers. Most of the current trials using CRISPR-based treatments are still in early stages. Although the potential uses of these technologies seem unlimited, their safe use in vivo has been limited by dependency on protein half-life, challenges in delivery, nonspecific activation of the immune system and engagement of off-targets (with an increase of adverse events) [6,9,10,11,12].

**Figure 1:**
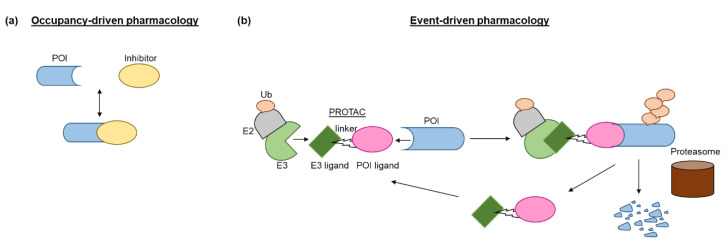
Different strategies of protein suppression. (**a**) A small-molecule inhibitor binds to the active site of a protein of interest (POI) to inhibit its enzymatic functions. Most enzyme inhibitors bind noncovalently and reversibly. (**b**) PROTAC directs E3 ligase to a POI and mediates the polyubiquitination of the POI by an E2 conjugating enzyme. The modified POI is recognized and degraded by the 26S proteasome. The PROTAC can then be recycled to induce the next round of the POI degradation. PROTAC: PROteolysis TArgeting Chimera; POI: protein of interest; Ub: ubiquitin.

Inspired by the fact that cells employ the ubiquitin-proteasome system (UPS) to maintain intracellular proteostasis, interest has focused on an alternative approach that aims to control protein function by regulating expression levels of a target protein rather than its activity. Such efforts have evolved from the discovery that proteasome inhibitors that block protein degradation have tumoricidal activity. Two examples approved for the treatment of multiple myeloma are carfilzomib and bortezomib [13]. Newer strategies involve the pharmacological hijacking of the cellular quality-control system to posttranslationally eliminate oncoproteins. Such a modality was first developed rather accidentially. Thalidomide, a drug historically more infamous than famous, was prescribed as a sedative to treat pregnancy-related morning sickness, which led to birth of thousands of children with severe defects in late 1950s/early 1960s [14,15]. After more than 60 years, the underlying molecular basis has been deciphered: thalidomide acts by promoting destruction of proteins needed for normal fetal development [15]. In the 1980s it gained a second life as an antiangiogenic drug, mitigating the growth of blood vessels in tumors. As such, it has been repurposed for treating multiple myeloma, and derivatives such as lenalidomide and pomalidomide have been developed to treat hematological malignancies. Thalidomide and its analogs are collectively referred to immunomodulatory drugs (IMiDs) and act as molecular glues that mediate ubiquitination of a protein of interest (POI) by promoting a protein-protein interaction between POI and an E3 ligase, cereblon (CRBN) being its direct binding target [16]. Thus, they lack affinity for the POI in the absence of the E3 ligase. Another modality that hijacks the UPS consists of PROTACs (PROteolysis TArgeting Chimeras). PROTACs have evolved from cell-impermeable peptide-small molecule chimeras to orally bioavailable compounds that tag unwanted proteins for destruction via ubiquitination in patients. They are heterobifunctional small molecules with two covalently linked ligands: one binds a POI, while the other one simultaneously recruits an E3 ubiquitin ligase. This transient binding event results in polyubiquitination of the POI and its destruction by the 26S proteasome. The PROTAC is then recycled to attack another copy of the POI, resulting in substoichiometric activity. This event-driven action is catalytic and eliminates the need to maintain high drug levels, both characteristics that distinguish PROTACs from classical occupancy-driven pharmacology of small-molecule inhibitors (Figure 1, Table 1). With the discovery of PROTACs, targeted protein degradation (TPD) expands the reach of drug development by enabling the degradation of targets that have been stigmatized as undruggable by traditional inhibitor-based tools. This progress has fueled the identification of additional TPD approaches that hijack endolysosomal and macroautophagic degradation pathways rather than the UPS system. While the proteasome-based technologies are limited to proteins with cytoplasmic domains, LYTACs (Lysosome TArgeting Chimeras) and AUTACs (Autophagy Targeting Chimeras)/ATTECs (Autophagy Tethering Compounds) promote the targeted destruction of extracellular proteins or entire organelles and protein aggregates, respectively. The non-UPS platforms are rather conceptual frameworks; much more biophysical/structural study is warranted. In the last years, other strategies for proximity-based therapeutic modalities beyond degradation have also been described including targeted phosphorylation using phosphorylation-inducing chimeric small molecules, targeted dephosphorylation and targeted deubiquitination followed by targeted protein stabilization [17,18,19].

In this review we will reflect on different targeted protein degradation approaches and focus on the current status of clinical translation of TPD in cancer treatment.

## 2. Targeted Protein Degradation Approaches

**Figure 2:**
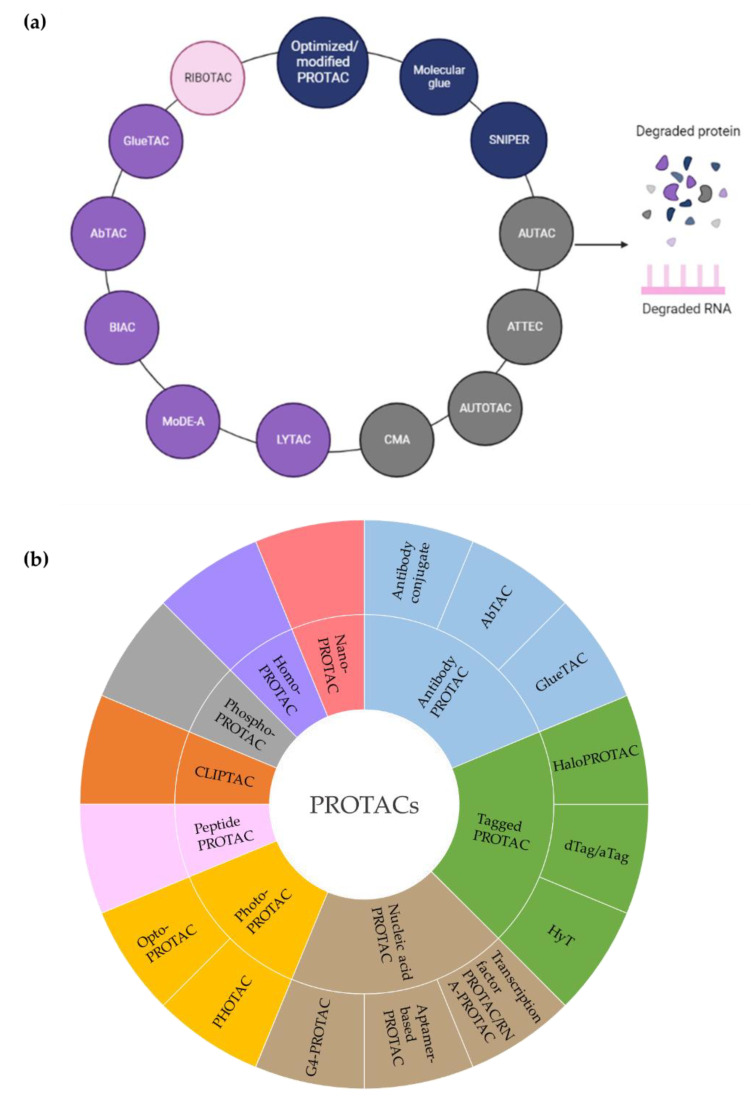
Overview of therapeutic modalities based on targeted degradation. (**a**) Targeted protein degradation approaches hijack either the ubiquitin-proteasome system or the autophagy or lysosomal pathways. RIBOTAC uses RNA-targeting small molecules and RNase L to eliminate intracellular oncogenic RNAs. Blue: ubiquitin-proteasome system; gray: autophagy pathway; violet: lysosomal pathway; pink: targeted RNA degradation. (**b**) Different PROTAC-based strategies are summarized. AbTac; antibody-based PROTAC; aTAG: AchillesTag; ATTEC: autophagy tethering compound; AUTAC: Autophagy Targeting Chimera; AUTOTAC: AUTOphagy TArgeting Chimera; BIAC: bispecific aptamer chimera; CLIPTAC: in-cell-click-formed proteolysis targeting chimera; CMA: chaperone-mediated autophagy; dTAG: degrader tag; GlueTac; GlueBody Chimera; G4:G-quadruplex; HyT: hydrophobic tagging; LYTAC: LYsosome TArgeting Chimera; MoDE-A; molecular degraders of extracellular proteins through the ASGPR; PROTAC: PROteolysis TArgeting Chimera; RIBOTAC: RIBOnuclease TArgeting Chimera; PHOTAC: PHOtochemically TArgeting Chimera; RNA: ribonucleic acid; SNIPER: Specific and non-genetic inhibitors of apoptosis protein (IAP)-dependent protein eraser.

Targeted protein degradation (TPD) has received a lot of attention as a novel and innovative chemical tool and therapeutic modality. By harnessing protein degradation pathways, TPD mediates depletion of a protein of interest from within or outside the cell (Figure 2, Table 2). Two main types of chemical degraders consist of (i) bifunctional molecules that carry a ligand binding to POI connected by a linker to another ligand binding to a component of the degradation machinery (e.g., the ubiquitin system, autophagy or the lysosomal system) and (ii) molecular glues that induce protein association of proteins that do not interact in the absence of these ligands [20]. TPD acts on a kinetic resolution, enabling near-complete target removal within minutes up to few hours [20]. Such progress expands the druggable space beyond small-molecule inhibitors.

### 2.1. Hijacking the UPS

**Figure 3:**
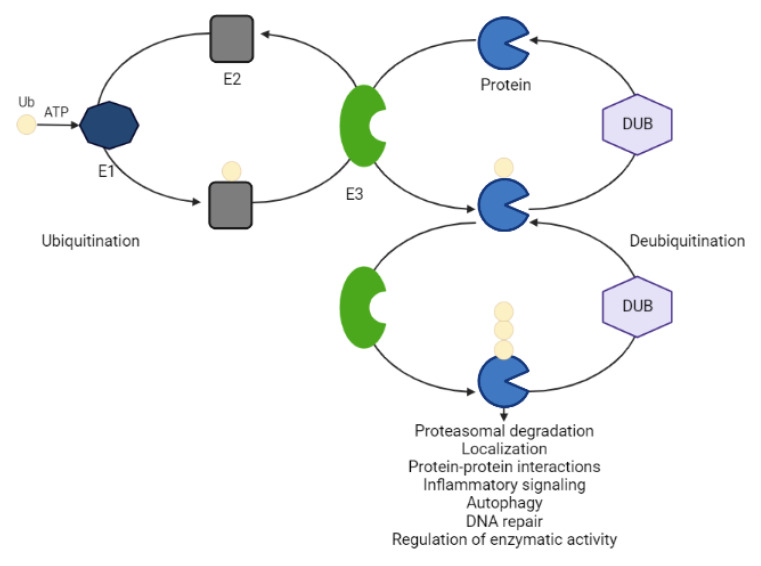
The process of ubiquitination and deubiquitination. Ubiquitin binds to the target protein through the sequential action of activating E1, conjugating E2 and ligase E3 enzymes at the expense of ATP. Deubiquitinating enzymes (DUBs) remove ubiquitin from the substrate by cleaving the isopeptide bond. The balance between ubiquitination and deubiquitination regulates a variety of cellular processes. ATP: adenosine triphosphate; Ub: ubiquitin.

The ubiquitin-proteasome system (UPS) is an essential pathway in the cell that processes the ablation of misfolded or damaged proteins involved in the pathogenesis of different diseases [21]. This tightly regulated process centers around an 8.6 kDA stable protein, so-called ubiquitin. The covalent attachment of ubiquitin to a lysine residue within the POI occurs in a concerted action of three ubiquitin enzymes (activating E1, conjugating E2, ligase E3) at the expense of ATP. These three enzymes act sequentially. Ubiquitin is first activated by E1 and then transferred onto E2. E3 interacts simultaneously with a ubiquitin-loaded E2 and the target POI by mediating isopeptide bond formation between the ubiquitin and a substrate lysine [22]. The structural variation of ubiquitin chains establishes a code that directs different cellular fates of the substrate (e.g., protein degradation, localization, protein-protein interactions, inflammatory signaling, autophagy, DNA repair and regulation of enzymatic activity) [22,23]. While K48-linked polyubiquitin chains mainly label proteins for 26S proteasome-mediated recognition and degradation, K63-linked polyubiquitination mainly signals for lysosomal degradation [21,24,25,26]. E3 ligases play a key role in the entire process of ubiquitination due to their specificity for substrates. There are approximately 1000 annotated E3 ligases that are categorized into the homology to E6AP C terminus (HECT) domain-containing E3s, the RING-between-RING (RBR) family E3s and the really interesting new gene (RING) finger domain-containing E3. Protein ubiquitination is a dynamic and reversible process (Figure 3). Deubiquitinating enzymes (DUBs) cleave the attached ubiquitin moieties from substrates, preventing degradative pathways [23]. In the human genome, more than 100 functional DUBs have been identified, which can be divided into eight families: ubiquitin-specific proteases (USPs), ubiquitin C-terminal hydrolases (UCHs), ovarian tumor proteases (OTUs), Jab1/MPN domain-associated metallopeptidase (JAMM) domain proteins, Josephin or Machado–Joseph disease protein domain proteases (MJDs), the monocyte chemotactic protein-induced protein (MCPIP) family, the motif interacting with Ub-containing novel DUB family (MINDY), and Zn-finger and UFSP domain proteins (ZUFSPs). DUB modifications have been implicated in tumorigenesis at multiple levels and inhibitors targeting DUBs are attracting increased attention from pharmaceutical companies [23].

Active ubiquitination and degradation of tumor suppressors (e.g., p53) are at the root of maintaining cancer cell proliferation. Here, targeted protein stabilization (TPS) instead of degradation would prove therapeutically beneficial [27]. Recently, heterobifunctional small molecules consisting of a recruiter of a DUB linked to a protein-targeting ligand were constructed to stabilize the levels of specific proteins that are otherwise actively degraded in a ubiquitin-dependent manner [19]. The proof of concept for the DUBTAC (deubiquitinase-targeting chimeras) platform has been provided in human cystic fibrosis bronchial epithelial cells and in hematoma cells. While its clinical translation remains to be seen, there are many fields other than cancer that could benefit from targeted deubiquitination and stabilization, including immunooncology, diabetes, Gaucher’s disease or Parkinson’s disease [19]. In diseases caused by haploinsufficiency, where loss of one gene copy is responsible for the pathology, DUBTACs may slow down the turnover rate of the POI to elevate its levels to attenuate disease progression [19].

#### 2.1.1. PROTACs (PROteolysis TArgeting Chimeras)

The PROTAC technology has experienced three generations of development. The first generation required peptide sequences for E3 ligase recognition and cell penetration. The first PROTAC was applied in Xenopus egg extract to target methionine aminopeptidase 2 to the Skp1-Cullin-F box complex for ubiquitination and proteasomal degradation [28]. The same approach was further employed to ubiquitinate and deplete the androgen and estrogen receptors (AR and ER), two cancer-associated targets involved in the progression of prostate and breast cancers, respectively [29]. Another peptide-based PROTAC engaged the von Hippel-Lindau (VHL) as an E3 ligase to degrade POI in intact cells [30]. However, these chimeric compounds exhibited low cellular activity at least partially due to poor cell permeability and chemical stability, limiting their clinical applicability. The second generation of PROTAC sought to design non-peptidic E3 ligase ligands to pave the ground for all small-molecule PROTACs. This concept succeeded by development of a compound consisting of a selective AR modulator tethered to nutlin, which is a ligand for the E3 ligase MDM2 and hinders MDM2 binding to its substrate p53 [31]. This PROTAC recruited AR to MDM2 for ubiquitination and degradation in HeLa cells. Despite advances in degradation efficiency, there are still some limitations including potential off-target specificity, high molecular weight and cytotoxicity. The third generation focuses on controllable PROTAC including phosphate-dependent and light-controlled PROTACs, which stimulate protein degradation via activated kinase signaling signal or visible light, respectively [32,33,34,35,36], and will be discussed in depth below.

**Figure 4:**
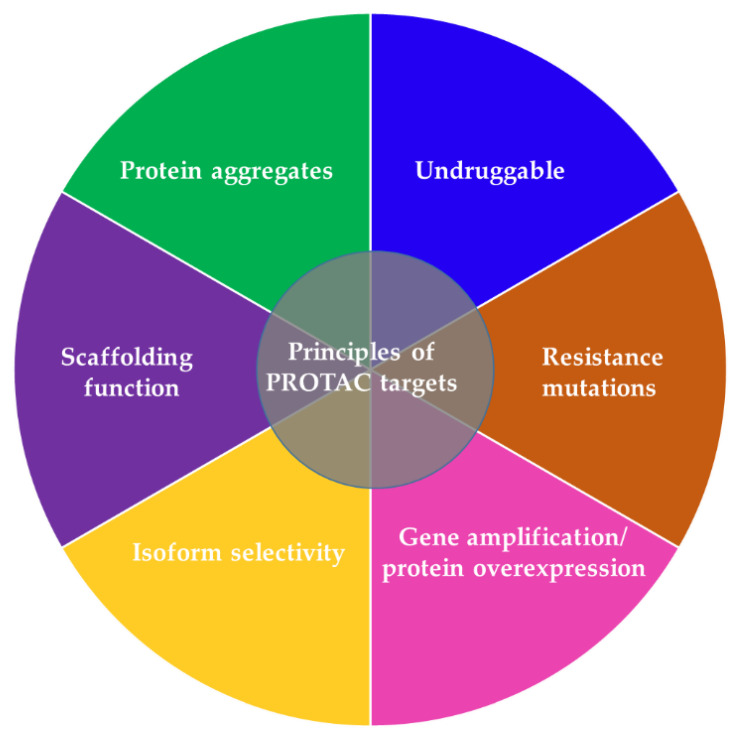
Overview of main targets of PROTAC degraders.

PROTACs have potential advantages compared to other traditional approaches to suppressing a POI, providing them with a greater chance for faster clinical development against a wider range of targets in multiple cancer types (Table 1, Table 2, Figure 4) [37,38]. Due to the catalytic mode of action (as reflected in the continuous and rapid ablation of the POI) PROTACs are required at significantly lower concentrations (in a nanomolar or picomolar range) to exert a biological effect and unlike traditional small-molecule inhibitors, they tolerate low affinity binding to the POI [37]. Previous reports thus suggested that ineffective inhibitors with weak kinase interactions may still have clinical relevance in PROTAC design and application [37,38,39]. PROTACs exhibit higher tissue and target selectivity toward mutant over wild-type protein both in vitro and in vivo (Figure 4), and thus provide a promising modality to treat cancer and other diseases [16,37,38]. Another advantage of PROTACs is their enhanced protein isoform selectivity (Figure 4). Winter and colleagues described selective destruction of the cell cycle kinase CDK6 (cyclin-dependent kinase 6) by a PROTAC while sparing its close homolog CDK4 [40]—a feature that distinguishes PROTAC treatment from approved CDK4/6 kinase inhibitors that fail to discriminate between these two kinases. Similar observations were reported on isoform specific SGK3 (serum/glucocorticoid regulated kinase family member 3) PROTAC degrader over SGK1/2 [41]. Both studies exemplify the benefit of the PROTAC approach in targeting kinase signaling pathways with enhanced efficacy and selectivity, more than is possible with conventional inhibitors [40,41]. Selectively attacking oncogenic proteins in diseased cells while sparing their homologs in healthy tissue may help reducing the off-target effects in patients. Linker composition and E3 complex/POI interactions that create a favorable ternary complex may at least partially explain wild-type/mutant and homolog specificity of PROTACs. Future studies will shed light on the exact mechanisms of selectivity evident for degraders.

Unlike small-molecule inhibitors that function via binding to catalytic or allosteric sites, PROTACs derived from a binder (e.g., peptide, chemical entities, etc.) on any POI surface pocket with reasonable affinity may cause target destruction [37,38]. This distinct mechanism of action allows PROTACs to attack clinically relevant targets that are traditionally considered undruggable such as transcription factors lacking catalytic sites for small-molecule inhibitors (Figure 4). PROTACs are able to mitigate not only catalytic activity of target enzymes but also eliminate other functions of the POI such as scaffolding functions that often remain intact despite attenuated enzymatic activity (Figure 4) [38]. This is of potential clinical relevance because unimpaired scaffolding functions are prone to induce rewiring of signal transduction which damages efficacy and manifests as resistance as seen with small-molecule inhibitors [38]. Of clinical importance, PROTACs can offer a thorough elimination of multi-protein complexes because they degrade both the target and the interacting subunit, whereas small-molecule inhibitors often attenuate the function of a target POI while sparing others [38,42,43].

PROTACs are also able to eradicate mutated targets (Figure 4). In fact, they can bypass acquired resistance to kinase inhibitors by targeting an alternative ligand-binding site. This is exemplified by the finding that by targeting an alternative RAS-binding domain of BRAF, a rigosertib-derived PROTAC promotes degradation of mutant BRAF and overcomes the mutation-mediated drug resistance [44]. Although administration of a PROTAC at a lower dosage mitigates the likelihood of resistant mutations emerging in the target, PROTAC treatment is still prone to elicit resistance. Causative alterations are not limited to the neo-substrates but can also arise within the E3 ligases [40,45,46,47,48]. To date the majority of reported PROTACs use either CRBN or VHL as the hijacked E3 ligase, with >30 proteins being destructed through CRBN and >20 via VHL [49,50,51,52]. Acquired resistance to these two E3 ligases has been reported in cancer cells following chronic treatment [48]. Unlike many targeted therapeutics, resistance did not result from secondary mutations that influence compound binding to the target POI but it was primarily caused by genomic alterations within the core components of the degradation machinery. In line with this, Winter and colleagues recently established a haploid genetics-based pipeline to profile the landscape of resistance mechanisms to small-molecule degraders [47]. They deciphered functional hotspots in the E3 ligases CRBN and VHL that tumor cells can use to evade degrader molecules. In fact, a number of identified hotspots were disrupted in patients that relapsed from degrader treatment, supporting the potential clinical relevance of their assay. This pipeline only covers copy number loss and splicing defects and mimics the scenario of homozygous mutations, whereas patients may harbor heterozygous mutations. Future data on clinical trials of degraders will provide additional insight into clinically relevant functional hotspots. A critical step in PROTAC development is the formation of a stabile ternary complex: although CRBN and VHL have a wide palette of substrates, some protein-ligase pairs do not endure long enough for the E3 ligase to ubiquitinate [53]. This however could be ensured by alternative E3 ligases. Among those are MDM2, DCAF15, DCAF16, RNF4, RNF114, FEM1B, KEAP1, AhR, cIAP1 and XIAP, which pose several distinct advantages including specificity for tissue, tumor, cell type or cell state, and synergistic tumoricidial effects through activation of pro-apoptotic cell cycle regulatory proteins [54].

**Figure 5:**
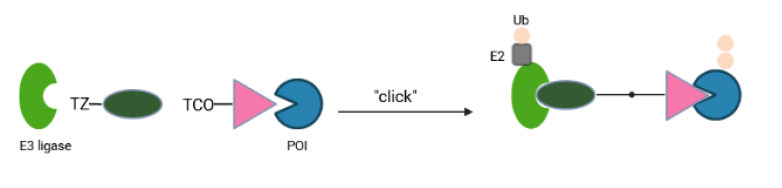
Schematic diagram of CLIPTACs. CLIPTACs consist of cell permeable precursors that join together via click-chemistry to form an active molecule in cells. POI: protein of interest; TCO: *trans*-cyclooctene; TZ: tetrazine; Ub: ubiquitin.

Although protein degradation has revolutionized drug discovery platforms, current PROTACs come with several shortcomings that are likely to restrict their potential as therapeutics. These heterobifunctional molecules require linker optimization, possess high molecular weight and a high polar surface area that is normally associated with poor cellular penetration, solubility and other drug-like properties. An alternative has been proposed using the idea of CLIPTACs (in-cell-click-formed proteolysis targeting chimeras) in which two small precursor molecules with the ability to click intracellularly will pass through cellular membranes more easily than one large compound (Figure 5). Heightman and colleagues generated a tetrazine-tagged E3 ligand and a *trans*-cyclooctene-tagged POI ligand as the precursors [55]. Via the click reaction between tetrazine and *trans*-cyclooctene, forming a covalent six-membered ring moiety, these two precursors created integrated PROTACs (CLIPTAC) in cells that successfully depleted oncogenic BRD4 or ERK1/2 in a CRBN E3 ligase- and proteasome-dependent manner [55]. The main advantage of CLIPTAC is a significant reduction of the molecular weight and polar surface area of the separate partners compared to the pre-assembled PROTAC molecule. Tuning the click reaction to proceed at a slower rate may help to avoid the possibility that the bioorthogonal combination reaction occurs outside the cells, resulting in a heterobifunctional molecule with high molecular weight and polar surface area that fails to penetrate into cells.

High bioavailability can be achieved by orally bioavailable prodrug PROTACs derived from CRBN ligands [56]. Wei et al. reported the first prodrug PROTAC based on the structure of CDK4/6 inhibitor ribociclib’s derivative, with the oral bioavailability up to 68% [56]. In melanoma cells, the degrader could not only degrade CDK2/4/6 simultaneously and effectively, but also induced cell cycle arrest and apoptosis of malignant cells. Another tool to increase bioavailability is offered by computer-aided drug design softwares to in silico predict the solubility and ADMET (Absorption, Distribution, Metabolism, Excretion and Toxicity) properties of molecules before design [51]. Furthermore, the cell/tissue permeability of PROTACs may be enhanced by using long flexible linkers to generate intramolecular hydrogen bonds to at least partially reduce polarity [57]. A further way is to attach cell-permeable peptides to E3 ligands [58]. Finally, identification of highly specific POI/E3 ligands is crucial for the design and development of potent PROTACs with minimal off-target effects.

**Figure 6:**
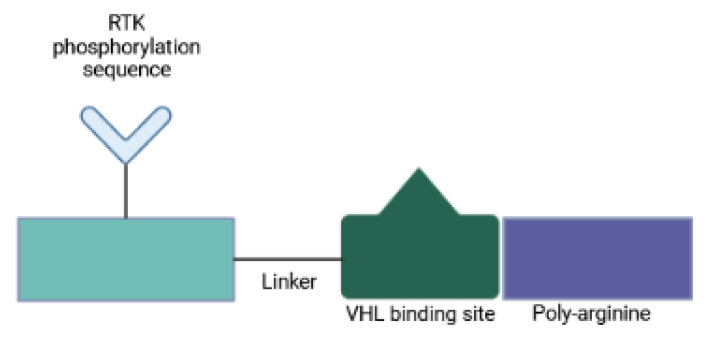
Schematic diagram of phosphoPROTACs. PhosphoPROTAC consists of an RTK phosphorylation sequence, VHL binding sequence and a poly-D-Arginine sequence connected by a linker. When the RTK is activated, the tyrosine of the RTK phosphorylation sequence is phosphorylated, recruits and activates POI with PTB and SH2 domains. The peptidic VHL binding sequence following hydroxylation of the proline residue recruits the VHL E3 ligase to mediate polyubiquitination and degradation of the POI, thereby inactivating tyrosine kinase signaling. RTK: receptor tyrosine kinase; VHL: von Hippel-Lindau.

Since PROTACs operate in a catalytic manner and enable systemic protein knockdown, their off-target toxicity is a major concern. One strategy to circumvent systemic undesired effects involves the use of designed peptides, so-called phosphoPROTACs, that can be conditionally activated via phosphorylation by specific growth-factor stimuli [32]. A recent study has described two phosphoPROTAC molecules that coupled the tyrosine phosphorylation sequences of the nerve growth factor receptor, TrkA, or the neuregulin receptor, ErbB3, with a peptide ligand for the VHL E3 ligase [32] (Figure 6). These phosphoPROTACs then recruited either the neurotrophic signaling effector FRS2α or the survival promoting PI3K, respectively, to be ubiquitinated and depleted upon activation of specific receptor tyrosine kinases and phosphorylation of the phosphoPROTACs. Anticancer properties were demonstrated both in vitro and in vivo. Activation of phosphoPROTACs was dependent on their kinase-mediated phosphorylation: (i) phospho-null variants remained inactive; and (ii) stimulation of unrelated growth factor receptors did not induce target protein knockdown. This approach provides not only a time- and dose-dependent control but also cell type selectivity, all features that distinguish phosphoPROTACs from nucleic acid-based strategies. Furthermore, conditional activation makes phosphoPROTACs suitable for the selective treatment of malignant cells. Another strength is that it is not likely to arouse drug-resistant mutants [32]. Current tyrosine kinase inhibitors mitigate cell proliferation by blocking enzymatic function, which in turn signals for a selective pressure for target mutations that evade inhibitor binding and preserve kinase activity. PhosphoPROTACs on the other hand require dysregulated kinase function to stop cellular signaling and tumor growth. Therefore, changes within the kinase structure to prevent phosphoPROTAC activation are anticipated to cause loss of kinase signaling too [32]. By changing the autophosphorylation sequence of a phosphoPROTAC, other receptor tyrosine kinase/effector pairings can be investigated.

**Figure 7:**
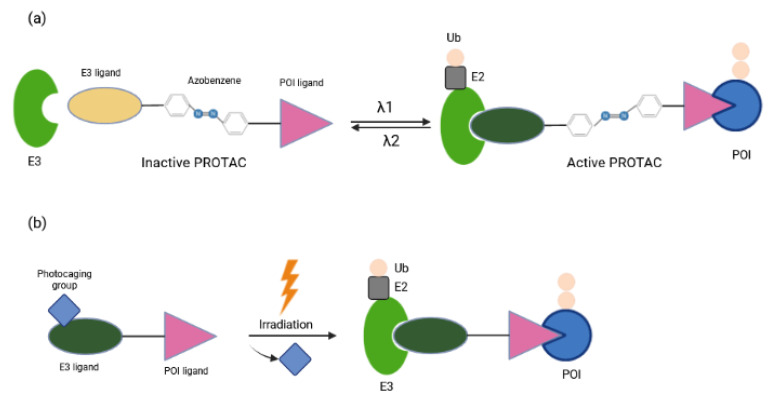
Schematic diagram of photochemical controls of PROTAC. (**a**) The PHOTAC molecule switches between an inactive (orange) and an active form (dark green) under blue-violet light. (**b**) The photocaged-PROTAC is activated in a UVA-dependent manner. The photocage group is then released, enabling the degradation of POI in a controllable manner. POI: Protein of interest; Ub: Ubiquitin.

Other approaches for tissue/cell specific degradation of POIs by PROTAC while avoiding deleterious effects elsewhere employ light stimuli [59,60]. Optical control can be achieved with caged compounds [61], with genetically engineered photoreceptors [62], or with synthetic photoswitches whose activity can be changed through a combination of photochemical isomerization and thermal relaxation [63,64]. Recently, several reports have focused on light-induced control of protein destruction [34,35,36]. By incorporating azobenzene photoswitches into PROTACs, Trauner and colleagues have described a novel strategy to control TPD with the spatiotemporal precision that light provides [34]. These so-called PHOTACs (PHOtochemically TArgeting Chimeras) are trifunctional molecules compromised of a ligand for an E3 ligase, a photoswitch and a ligand for POI (Figure 7a). They are inactive as degraders in the dark and become active under blue-violet light (380–440 nm). Activated PHOTACs gradually lose their activity through thermal relaxation, or can be quickly inactivated photochemically [34]. Thus, their inactivation is much less dependent on dilution, clearance, or metabolism. As a proof of principle they were used to tame BET (Bromo- and extra-terminal) family proteins or FKBP12 by binding CRBN E3 ligase complex and mediating proteolysis in a light-dependent manner, which in turn suppressed viability of lymphoblastic leukemia cells. The concentrations needed for maximum photo effect were in the nanomolar range, minimizing possible off-target effects. Another strategy for light-inducible control of PROTAC activity has been provided by Wei and colleagues [36]. In the dark, nitroveratryloxycarbonyl (NVOC) labeling on the CRBN E3 ligand as photolabile blocking group hindered the association between PROTAC and E3 ligase, whereas upon UV irradiation the photocaging group was released from PROTAC, facilitating the formation of the POI-PROTAC-E3 complex (Figure 7b). These so-called opto-PROTAC molecules were applied for restricted degradation of cancer-related proteins of IKZF1/3, BRDs and ALK fusion proteins at a specific time and rate by UVA illumination. Tumoricidal properties were reflected in reduced cancer cell proliferation in an optically-controlled manner. Despite their advantages, photoPROTAC technologies still come with some limitations. Considering UVA irradiation might cause DNA damage and is mainly applied in blood, skin and lung cancers due to its inefficiency in penetrating tissues, it would be more appropriate to develop alternative methods that do not require UVA exposure [36]. Caging groups with absorption within the near-infrared region may enhance tissue penetration and improve clinical output. In vivo experiments using mouse models are definitely crucial to validate the functions of these molecules.

Conventional small-molecule PROTACs generally display unfavorable pharmacokinetics and lack of tumor specificity, which may contribute to systemic toxicity due to their nonspecific distribution in normal tissues. One way to achieve tumor specific delivery of PROTACs has been proposed by using gold nanoparticle (GNP)-based multi-headed PROTACs [65]. Cer/Pom-PEG@GNPs composed of interconnected ligands for POI and E3 ligase promoted effective ALK degradation in a dose- and time-dependent manner and hindered proliferation of a lung adenocarcinoma cell line with minor off-target toxicity. Although in vivo data is missing, Cer/Pom-PEG@GNPs as a nano-based drug carrier promises prolonged circulation and specific delivery of drugs to tumor regions, and can be beneficial in patients resistant to ALK kinase inhibitors [65]. Furthermore, Yu and colleagues reported polymeric PROTAC (POLY-PROTAC) nanotherapeutics for tumor specific targeted degradation [66]. The POLY-PROTACs self-assemble into micellar nanoparticles and sequentially respond to extracellular matrix metalloproteinase-2, intracellular acidic and reductive tumor microenvironments. The POLY-PROTAC nanoparticles carry azide groups for bioorthogonal click reaction-amplified PROTAC delivery to the tumor region. Tumor specific BRD4 depletion via the POLY-PROTAC nanoplatform combined with photodynamic therapy resulted in tumor regression in a mouse xenograft model of MDA-MB-231 breast cancer [66]. For the successful translation of the so-called nanoPROTACs into the clinical setting, safety analysis and optimization of a chemical and manufacturing control procedure are required [67].

**Figure 8:**
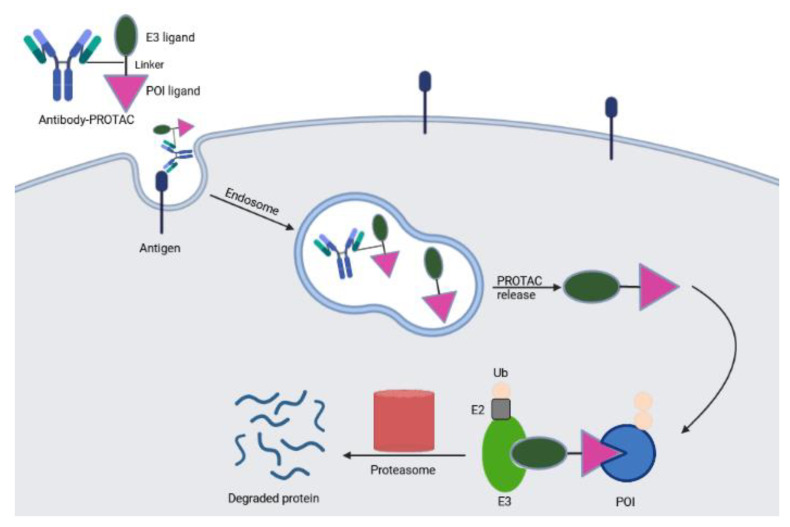
Schematic depiction of degrader-antibody conjugates. These molecules allow cell specific delivery of the degrader. Endocytosis of the conjugates mediates the release of active PROTAC which marks the POI for proteasomal destruction.

Selective delivery of a broad spectrum PROTAC into specific cell types is also feasible by an antibody-PROTAC conjugate. Maneiro et al. described a trastuzumab-PROTAC conjugate in which E3 ligase-catalyzed degrader activity is caged with an antibody linker which can be hydrolyzed upon antibody-PROTAC internalization, releasing the active PROTAC and stimulating protein destruction [68] (Figure 8). Proof of principle was provided by degradation of BRD4 only in HER2 positive breast cancer cells but not in HER2 negative background. The event required proteasome activity: incubation with bortezomib, a proteasome inhibitor, prevented antibody-PROTAC mediated BRD4 depletion. Other studies expanded the target spectrum and applied this concept to different tumor models [69,70,71,72].

#### 2.1.2. Tag-Based Chemical Degraders

**Figure 9:**
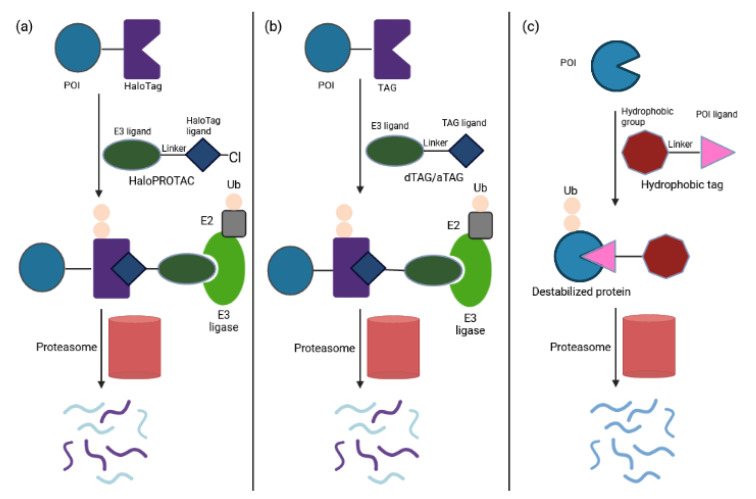
Schematic depiction of tag-based degraders. (**a**) The HaloPROTAC utilizes recognition of hexyl chloride tags and promotes destruction of HaloTAG-fused POI. The modality simplifies the optimization of PROTACs. (**b**) The dTAG molecule is composed of an E3 ligase ligand linked to a POI ligand, which facilitates formation a ternary complex between the fusion protein and E3 ligase, leading to polyubiquitination and degradation of the POI. The key difference between the dTAG and aTAG technologies is the identity of the TAG protein used. (**c**) Misfolded proteins are degraded through recognition of hydrophobic patches.

The selection of appropriate E3 ligase/E3 ligand systems is crucial for the progress of PROTAC research. Application of PROTACs in an endogenous setting is mainly limited to target proteins with available ligands. An advancing solution has been provided by tag-directed chemical degrader systems in which the tag-POI fusion protein was expressed in cells and the universal PROTAC was administered to attract the candidate E3 ligase and the tag of the tag-POI fusion [51]. Measuring the abundance of tag-POI complex validated whether the candidate E3 ligase could mediate POI destruction. The most-widely used tag-based strategies are HaloPROTAC (Figure 9a) and degrader tag (dTAG) (Figure 9b) [73,74,75,76]. HaloPROTACs degrade HaloTag7 fusion proteins by combining chloroalkane HaloTag ligands with a small molecule ligand for the VHL E3 substrate receptor, and exhibit favorable potency and kinetics [73]. Proof of concept was demonstrated by depletion of cytoplasmic proteins such as ERK1 and MEK1. Tovell et al. improved HaloPROTAC degrader probes by combining them with CRISPR-Cas9 technology [74]. The lead probe induced reversible depletion of two endosomally localized proteins, SGK3 and VPS34, thereby blocking downstream signaling.

The dTAG system uses PROTACs to degrade target proteins that have been genetically fused to a mutant isoform of FKBP12 [75,76]. Both CRBN- or VHL-recruiting dTAG molecules have been proven suitable for in vivo use. Recently, Fischer and colleagues reported a novel degradation tag BRD4_BD1_L94V along with the corresponding CRBN-based heterofunctional degrader utilizing a bump-and-hole approach [77]. They also proved the compatibility of simultaneous application of the BRD4_BD1_L94V system and the dTAG system. This strategy complements currently available degradation tags to attack disease co-dependencies.

Furthermore, Veits et al. recently reported a novel degradation methodology on fusing a POI to the small protein MTH1 (MutT homolog-1), which serves as a ligand-binding tag [78].This AchillesTag (aTAG) can be paired with different heterobifunctional degraders that recruit the CRBN E3 ligase into close proximity, leading to ablation of any aTAG-fused POI (Figure 9b). Proof of principle has been provided by selectively controlling Chimeric Antigen Receptor (CAR) protein levels [78]. Treatment with aTAG degraders attenuated CAR-mediated target tumor cell killing and T-cell activation/cytokine release. These effects were rapid and reversible as CAR-T activity was restored upon drug removal. Six CAR-T cell products are already in the market for the treatment of B-cell acute lymphoblastic leukemia, lymphomas and multiple myeloma: Kymriah (Tisagenlecleucel), Yescarta (Axicabtagene ciloleucel), Tecartus (Brexucabtagene autoleucel), Breyanzi (Lisocabtagene maraleucel), Abecma (Idecabtagene vicleucel) and Carvykti (Ciltacabtagene autoleucel). However, these therapies have been associated with unique adverse events including cytokine-release syndrome, neurologic events and immune effector cell-associated neurotoxicity [78,79]. Therefore, fine tuning of protein expression in CAR-T therapy provides clinical benefit. To date a suicide switch in the CAR has been proposed to improve the safety of T cell adoptive immunotherapy for lymphomas, which however resulted in the irreversible loss of treatment [80]. The aTAG degradation system was also efficacious in vivo with favorable pharmacokinetic properties. Altogether, the aTAG model provides another layer in the drug discovery efforts to address adverse consequences and define desired in vitro and in vivo properties of a degrader therapeutic.

Unfolded or misfolded proteins expose hydrophobic regions that signal for recruitment of E3 ligases to degrade unwanted POIs in a proteasome-dependent fashion. Several studies have mimicked protein unfolding by labeling specific targets with low molecular weight hydrophobic tags to promote their destruction [81,82,83,84,85]. This modality has been applied to degrade the pseudokinase Her3, an undruggable target implicated in breast, ovarian and non-small cell lung cancers, by bifunctional molecules consisting of a covalent Her3 targeting small molecule linked to a hydrophobic adamantane moiety [82]. The resulting silencing of Her3 hindered productive heterodimerization of Her3/Her2 and Her3/c-Met, and reduced proliferation of Her3-dependent cell lines. In addition, Ma et al. developed an EZH2 inhibitor-based degrader using hydrophobic tagging (HyT) strategy by linking an EZH2 inhibitor to a bulky adamantyl group to degrade EZH2 [84]. This target protein is the main enzymatic subunit of the PRCs complex; high expression levels are associated with poor prognosis in multiple types of cancer. Using hydrophobic tagging, the lead degrader compound selectively killed triple negative breast cancer cells in vitro and in vivo, whereas existing EZH2 inhibitors fail to do so [84]. Another hydrophobic tagged dimeric molecule was shown to reduce cellular levels of SRC-1, a transcription co-activator, which in turn suppressed cancer cell migration and invasion [83]. A further successful application of this approach has been demonstrated by Gustafson et al. [85]. Molecules containing hydrophobic degrons linked to small-molecule AR ligands induced AR ablation, decreased expression of AR target genes and attenuated proliferation of androgen-dependent prostate cancer cell lines. The toxic effects were similar to those seen with enzalutamide, an FDA-approved inhibitor of AR signaling. Remarkably, this drug also retained its antiproliferative properties in cells resistant to current standard of care drugs for castration-resistant prostate cancer [85]. Altogether, the hydrophobic tagging strategy (Figure 9c) adds to the emerging paradigm of targeted protein degradation as a therapeutic strategy. It holds the promise to develop peptide-based degraders with improved cell permeability and metabolic stability in order to manipulate disease-relevant proteins and drug-resistant mutants that have been insensitive to traditional approaches.

#### 2.1.3. Degrader Systems Based on PROTACs

##### Homo-PROTACs as Suicide Molecules

The human genome comprises approximately 1000 predicted E3 ubiquitin ligases that are important not only in normal cellular physiology but also in diseased states, making them attractive targets for drug discovery. Overexpression of E3 ligases is associated with poor clinical prognosis or drug resistance in cancer cells. These enzymes do not exhibit deep and druggable active sites for binding to small molecules; their inhibition is generally achieved by targeting protein-protein interactions. Only few potent compounds have been described that bind to the E3 substrate recognition site. However, competition with high-affinity endogenous substrates may increase unspecific cytotoxicity. Further, E3 ligases are multi-domain and multi-subunit enzymes [86]. Thus, targeting an individual binding site leaves other interactions functional, resulting in ineffective blockade of the enzyme. To overcome these obstacles, interest has focused on the design of bivalent compounds to dimerize an E3 ligase to trigger its suicide-type chemical knockdown inside the cells. The so-called homo-PROTACs are a unique type of PROTACs comprised by two identical molecules linked together. Proof of concept has been provided by several VHL- and CRBN-based homo-PROTACs with no in vivo therapeutic potency addressed [50,86,87,88].

Recently, He et al. extended this principle to MDM2, an oncogenic E3 ligase and antitumor target [89]. A homo-PROTAC for an effective disruption of MDM2-p53 interaction may lead to a distinct strategy in cancer therapy. The lead homo-PROTAC compound dimerized MDM2 with high binding activity and induced its proteasomal self-degradation [89]. As a consequence, p53 expression was increased in a dose-dependent manner, triggering apoptosis in A549 non-small cell lung cancer cells. In vivo tumoricidal activity was demonstrated in a xenograft mouse model, making this study the first example of a homo-PROTAC with in vivo therapeutic potency. This approach offers a new avenue to overcome the bottleneck of the dose-related adverse effects including the risk for hematological diseases [90,91] of MDM2-p53 small-molecule inhibitors observed during clinical studies.

##### Nucleic Acid-Based PROTACs

RNA-PROTACs

**Figure 10:**
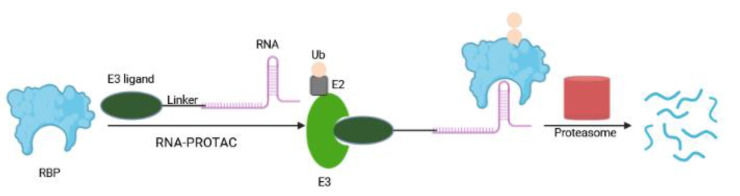
Schematic depiction of RNA-PROTACs. An RNA-PROTAC with a short oligonucleotide binds the RNA binding domain of RBP and directs it for degradation. RBP: RNA binding protein; Ub: ubiquitin.

A new type of PROTAC, so-called RNA-PROTAC, aims to degrade RNA binding proteins (RBPs), a class that until now has been proven difficult to target pharmacologically [92]. RBPs interact with target RNAs in a sequence- and structure-dependent fashion via their unique RNA binding domain. Their dysregulation (upon genetic alteration, epigenetic change, noncoding RNA-mediated regulation, and posttranslational modifications) are at the origin of many diseases ranging from neuronal disorders to cancers [93,94,95]. Altered RBPs influence several steps in the development and progression of cancer, including sustained cell proliferation, evasion of apoptosis, avoiding immune surveillance, inducing angiogenesis, and activating metastasis [93]. RNA-PROTAC chimeric structures employ small RNA mimics as targeting groups that dock the RNA-binding site of the RBP, whereby a conjugated E3-recruiting peptide derived from the HIF-1-alpha protein directs the RBP for proteasomal degradation (Figure 10). The first RNA-PROTACs target the stem cell factor and oncoprotein Lin28 and the splicing factor RBFOX1 in cancer cell lines [92]. This strategy has some shortcomings including the instability of RNA oligomer and the requirement of RNA secondary structure for its proper interaction with RBPs [96]. RNA-PROTACs have large molecular weights, making their clinical applicability challenging [97]. Here, cellular uptake may be improved by employing the CLIPTAC approach [55] to generate smaller RNA-PROTAC precursors. Furthermore, the synthesis of RNA-PROTACs and their permeability may be advanced by using water-soluble, non-peptidic linkers and E3 ligands; and application of nanoscale delivery systems may improve their efficiency in vivo [97,98].

Transcription Factor PROTACs

**Figure 11:**
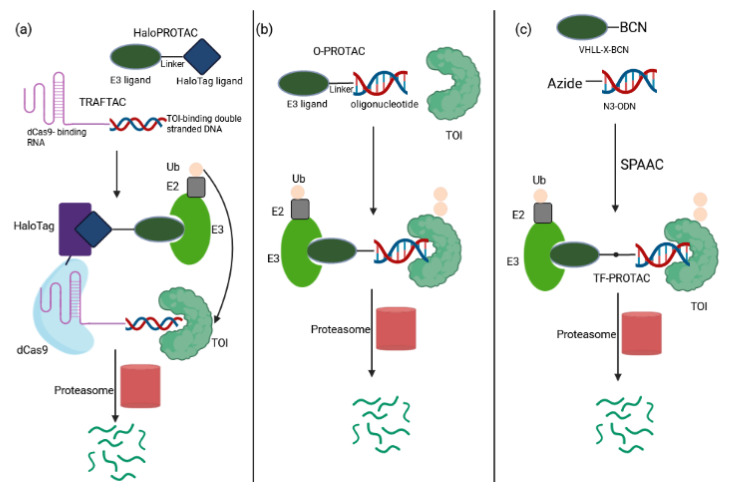
Overview of degradation of transcription factors. (**a**) TRAFTAC, a heterobifunctional dsDNA/CRISPR-RNA chimera, recruits E3 ligase complex through dCas9-HT7 in the presence of HaloPROTAC. TRAFTAC binds to dCas9-HT7 via its RNA moiety while dsDNA portion of the chimera binds to the transcription factor of interest (TOI). Addition of HaloPROTAC recruits VHL E3 ligase complex to the TOI, thereby targeting it for ubiquitination and proteasomal degradation. (**b**) In the O-PROTAC model the dsDNA is incorporated in the TOI binding ligand of the PROTAC. (**c**) A BCN-modified VHL ligand (VHLL-X-BCN) is incorporated onto an azide-modified DNA oligomer (N3-ODN) via a copper-free strain-promoted azide–alkyne cycloaddition (SPAAC) reaction, forming a TF-PROTAC to recruit the VHL E3 enzyme, thereby triggering ubiquitination and degradation of the TOI by the 26S proteasome. dsDNA: double-stranded DNA; Ub: ubiquitin.

Transcription factors (TF) are central in numerous diseases, yet remain incurable due to the lack of enzymatic activity and ligandable sites. In a recent study, Crews and colleagues have co-opted the DNA-binding capability of TF to develop TRAnscription Factor TArgeting Chimeras (TRAFTACs) [99]. The TRAFTAC is an oligonucleotide with a short, double-stranded DNA TF recognition sequence that can simultaneously bind to a transcription factor of interest (TOI) and VHL-E3 ligase via an intermediary HaloTag fused dCas9 protein (dCas9HT7) (Figure 11a). This multicomponent approach (a TRAFTAC, dCas9HT7 and a HaloPROTAC) resulted in proteasomal depletion of two disease-causing TFs: NF-κB (a key player in cell proliferation and overactivated in many cancers and inflammatory diseases) and brachyury (involved in tumor migration, invasion, and metastasis, and not expressed in normal adult human cells). In vivo efficacy was proven in a zebrafish model. Similar to PROTACs, TRAFTACs exhibit an event-driven pharmacology, and require a brief interaction of TOI with the chimeric oligo to induce TF destruction in a catalytic fashion. The multicomponent complex can bind to another TOI molecule after completing the first ubiquitination cycle. These features and their increased in vivo stability distinguish TRAFTACs from oligonucleotide-derived decoy elements [100]. Thus, the TRAFTAC strategy offers a creative tool to advance drugging TFs with a known DNA-binding sequence via the engagement of endogenous substrate mimics [99,100]. A major hurdle of the multicomponent nature is the limited bioavailability. The success of its utility relies on the ectopic expression of the Cas9 protein in cells. An efficient delivery strategy may enhance the translational possibilities for TRAFTACs in patients [99,100].

A complementary drug discovery and development platform has been proposed by the O-PROTAC model, in which a double-stranded oligonucleotide is incorporated as a TOI binding moiety in PROTAC [101] (Figure 11b). This modality has been successfully applied to destruct ERG, a TF overexpressed in 50% of both primary and metastatic prostate cancer [102], and LEF1, another cancer-related TF involved in migration and invasion, with potent efficacy in cultured cells. O-PROTAC offers straight-forward predictability, reprogrammability and superior stability.

The field evolves at a rapid pace as highlighted by a recent report of TF-PROTAC that links an DNA oligonucleotide to an E3 ligase ligand via a click reaction, to selectively degrade the TOI (Figure 11c). Here, commercially available azide-modified DNA oligomers are conjugated to the bicyclooctyne (BCN)-modified VHL ligands with various linkers (VHLL-X-BCN) via a copper-free strain-promoted azide–alkyne cycloaddition reaction [96]. The selectivity of TF-PROTACs depends on the DNA oligonucleotides used that can be specific to the TOI. Proof of concept has been demonstrated by targeted degradation of two cancer-relevant TFs, NF-κB and E2F, in a VHL E3 ligase and proteasome-dependent manner, thereby inhibiting cellular proliferation.

Altogether, compared with RNA, the DNA oligomer is more stable and the DNA binding specificity of TFs is better defined than the RNA binding specificity of RBPs. Therefore, TF-targeting platforms expand the druggable target spectrum with therapeutic benefits for patients with cancer and other diseases.

G4-PROTACs

G-quadruplexes (G4s) are four-stranded nucleic acid structures of DNA or RNA rich in guanine bases that are enriched in gene promoter regions. Many G4s harbor physicochemical and structural properties that render them favorable for drug design. Transcriptional repression of pathogenic proteins through stabilization of G4 structures or telomerase inhibition by telomeric-G4s have been suggested as novel antitumor strategies; a first-in-class G4-interacting compound has reached phase II trials in neuroendrocine/carcinoid tumors (NCT00780663) [103]. Recently, Patil et al. reported the use of G4 as a PROTAC warhead to CRBN and VHL small molecule ligand, respectively, for targeted degradation of a G4-binding protein (RHAU/DHX36) in HeLA and K562cancer cell lines [104].

Aptamer-PROTACs

Aptamers are single-stranded DNA or RNA oligonucleotides that bind to the target protein with high specificity and affinity. They possess favorable in vivo safety profiles without potential immunogenicity, establishing them as targeted therapeutics in oncology [105,106,107,108]. Sheng and colleagues described a strategy for modifying PROTACs with an aptamer to overcome the limitations of conventional PROTACs such as cell type selectivity [109]. The first aptamer-PROTAC was designed by conjugating a BET-directed PROTAC to the nucleic acid aptamer AS1411 via a cleavable linker. Compared to the unmodified BET PROTAC, the designed molecule improved tumor targeting specificity, leading to enhanced in vivo BET degradation and antitumor potency in a breast cancer xenograft model, and reduced toxicity. Hence, this technology holds the promise to improve the drug-likeness of conventional PROTACs.

Recently, Tan and colleagues provided the first proof of concept evidence using nucleic acid aptamer as a targeting ligand [110]. The designed molecule ZL216 promoted the formation of nucleolin-ZL216-VHL ternary complex by using AS1411 as a ligand for binding to nucleolin, which potently eliminated nucleolin in breast cancer cells in vitro and in vivo, and inhibited proliferation and migration of breast cancer cells in vitro [110]. Although ZL216 confers enhanced water solubility and tumor-selective binding, the therapeutic potential and pharmacokinetic features in vivo require further evaluation.

#### 2.1.4. Molecular Glues

**Figure 12:**
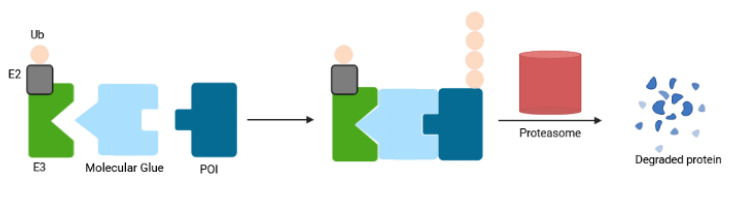
Schematic depiction of molecular glue degraders. These molecules acts as a protein-protein-interaction inducers to enhance or stimulate interactions between E3 ligase and the POI, thereby triggering ubiquitination-mediated proteasomal degradation of the POI. Ub: ubiquitin.

Molecular glue degraders are small, drug-like monovalent compounds that induce interactions between an E3 ligase and a target protein, promoting the degradation of the latter in a proteasome-dependent manner [111] (Figure 12). Unlike PROTACs, these molecules lack a linker within their structure, resulting in lower molecular weight. Molecular glues do not depend on a binding pocket on their target for action, making them suitable for depletion of undruggable proteins. The microbial products rapamycin, FK506 (tacrolimus) and cyclosporin A (sandimmune) with immunosuppressive properties are among the first molecular glues described [112,113]. Auxin represents another example for natural monovalent degraders. This pivotal phytohormone involved in plant growth and development mediates TIR1 ubiquitin ligase-catalyzed degradation of the AUX/IAA family of transcription repressors [114]. The auxin-inducible degron (AID) system enables its applicability in nonplant cells [115]. However, the need to express TIR1 and the enhanced risk of immunogenicity when engineered in mammalian cells limit its therapeutic potential.

Similarly, the clinically approved thalidomide and its analogs known as immunomodulatory drugs (IMiDs) exert their therapeutic activity via a molecular glue mechanism by reprogramming the target spectrum of the E3 substrate receptor CRBN. IMiD binding to CRBN resulted in proteasomal depletion of the IKAROS family members IKZF1 (Ikaros) and IKZF3 (Aiolos) that are the lymphoid transcription factors crucial in myeloma cell survival [116,117,118]. Lenalidomide eliminated myelodysplastic syndrome cells with deletion of chromosome 5q by promoting the ubiquitination and degradation of CK1α [119]. Several cellular proteins have been unraveled to be potential neo-substrates of IMiDs, which may facilitate development of novel IMiDs [15,120,121]. IMiDs have been also modified to generate chemically inducible degradation systems [122,123,124]. When tagged with an IMiD degron, drug treatment induced rapid CRBN-dependent destruction without the need for an exogenous ubiquitin ligase, as is required by the AID system [122,123,124]. In vivo potency was demonstrated with the IKZF3 degrons [122,123]. This IMiD-dependent inducible system has been applied to control CAR-T cell therapy in mice. A mouse xenotransplant model for acute lymphoblastic leukemia verified the ability of a degron-tagged CAR to target and kill CD19-positive cells, triggering complete control/clearance of the tumor. The activity of CAR19-degron could also be regulated in vivo by dosing the clinically approved lenalidomide [122]. These results indicate that degron tagging will not only allow on/off switching of CAR-T activity, but also that in vivo fine tuning of CAR-T activity could be feasible by adjusting lenalidomide dosing or dose regimen over the course of CAR-T therapy. This strategy thus holds the promise to mitigate potential toxicities associated with CAR-T therapy as described above.

Similar to the IMiD pharmacology, the arylsulfonamide indisulam and its analogs E7820 and tasisulam function via a molecular glue mechanism to drive the degradation of the essential RNA-binding protein RBM39 and the closely related splicing factor RBM23 (because of the high sequence conversation between their RBM domains involved in indisulam-induced molecular recognition) by chemically reprogramming the substrate DCAF5 [125,126,127]. The majority of molecular glues have been discovered serendipitously for a given target. Recent studies report experimental strategies for a rational design of such compounds, revealing a series of novel β-catenin and cyclin K degraders among others [47,128,129,130].

Despite the therapeutic efficacy of drug-induced destruction of transcription factors and other cancer targets, a subset of proteins remain resistant to targeted degradation using existing approaches. An alternative mechanism has been described in which a small molecule induced the highly specific, reversible polymerization of a POI, followed by its sequestration into cellular foci and subsequent depletion [131]. The small molecule BI-3802 induced formation of BCL6 filaments which led to ubiquitination of BCL6, a transcriptional repressor critical for the tumorigenesis of germinal center B cells, by the E3 ubiquitin ligase SIAH1, thereby triggering its degradation [131]. Recently, another BCL6-degrader with tumoricidal activity in a lymphoma xenograft mouse model following oral dosing has been reported but the mode of degradation has not been deciphered [132].

Altogether, molecular glue degraders open highly appealing avenues for the development of antitumor therapeutic agents and synthetic biology.

#### 2.1.5. SNIPERs

Specific and non-genetic inhibitors of apoptosis protein (IAP)-dependent protein erasers (SNIPERs) are chimeric molecules designed to induce IAP-mediated ubiquitination and proteasomal degradation of POI. They recruit the IAP family of RING-type E3 ubiquitin ligases—cIAP1, cIAP2 and XIAP with antiapoptotic properties. Cancer cells often overexpress IAPs to evade apoptosis with a concomitant increase in resistance to cancer therapy, making these proteins attractive drug targets. The chemical structure of a SNIPER consists of a selective IAP antagonist such as bestatin, MV1 and LCL161, a PEG linker and a peptide- or small-molecule-based POI specific component [133,134,135]. Unlike the chimeric molecules that recruit CRBN and VHL ligases, SNIPERs promote simultaneous destruction of IAPs along with the target protein, which may display synergistic effects on induction of apoptotic cell death [136]. Among the target proteins successfully depleted by SNIPERs at nanomolar concentrations are AR, ER, BCR-ABL, BRD4, Notch I and others [133,134].

### 2.2. Hijacking the Non-UPS

#### 2.2.1. (Macro)autophagy Degradation Targeting Chimeras

**Figure 13:**
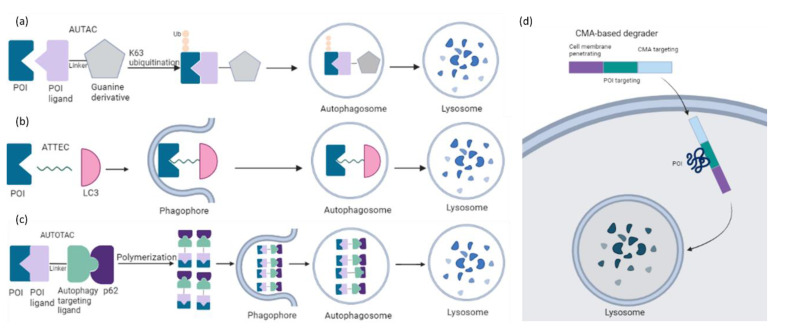
Concepts of degrader technologies hijacking autophagy pathways. (**a**) AUTACs bind to the POI and attach a degradation tag mimicking S-guanylation, a posttranslational modification that triggers K63 ubiquitination of the POI. The POI is recognized by the autophagy receptor SQSTM1/p62 and is recruited to the selective autophagy pathway for depletion. (**b**) ATTECs interact with both the POI and LC3, tethering the POI to the phagophores or autophagosomes for subsequent autophagic degradation. (**c**) The central mode of action in AUTOTAC is the ability of the p62-binding ligand to induce a conformational activation of otherwise inactive p62 into an autophagy-compatible version. Upon binding to the p62 moiety, p62 exposes PB1 and LIR domains, promoting p62 self-polymerization in complex with targets and its interaction with LC3 on autophagic membranes. (**d**) The CMA-based degrader first enters the cell, then binds the target protein via the POI binding sequence and is then transported to the lysosome for degradation. POI: protein of interest; Ub: ubiquitin.

The capacity of the dominating TPD platforms such as PROTAC is limited to soluble intracellular proteins because of their dependence on the UPS. Harnessing an alternative intracellular degradation mechanism such as macroautophagy may broaden the target spectrum including aggregated proteins, non-protein biomolecules, and organelles. The first autophagy-mediated degraders are chimeric molecules called AUTACs (Autophagy Targeting Chimeras), that consist of a S-guanylation-inspired degradation tag and a specific binder of an intracellular target of interest [137,138] (Figure 13a). Of note, S-guanylation is still dependent on K63-linked ubiquitination of the target. AUTAC degraded not only proteins but also fragmented mitochondria. Mitochondria-targeted AUTAC accelerated both the removal of dysfunctional fragmented mitochondria and the biogenesis of functionally normal mitochondria in Down Syndrome-derived fibroblast cells. The generality of the modality has been demonstrated by specific AUTACs against methionine aminopeptidase 2, FKBP prolyl isomerase 1A and BET family proteins followed by target clearance in HeLa cells. These data open a new window in research on autophagy-based agents with cargo specificity. Its applicability in cancer treatment requires further investigation.

A complementary approach was proposed by autophagy tethering compounds (ATTECs) to target non-protein biomolecules or relevant organelles [139,140]. These glues tether the POI with autophagosomes through direct binding to the POI and the key autophagosome-associated protein LC3 (Figure 13b). Li et al. have described that ATTECs against mutant huntingtin (mHTT) directed the protein to the autophagosome for clearance in vitro and in vivo, thereby rescuing phenotypes associated with Huntington’s disease [139]. The autophagy activity per se remained unchanged. A recent study reported autophagic degradation of lipid droplets (LD) via ATTEC targeting [140]. LDs are lipid-storing cellular structures, which are abnormally accumulated in many diseases. LD-ATTEC compounds were generated by connecting LC3-binding molecules to LD-binding probes via a linker. Their application resulted in almost complete clearance of LD and rescued LD-associated phenotypes in cells and in two independent mouse models with hepatic lipidosis. This study provides evidence that all autophagy substrates could be targeted for degradation by designed ATTECs.

Another chemical platform called AUTOphagy TArgeting Chimera (AUTOTAC) employs bifunctional molecules composed of target-binding ligands linked to autophagy-targeting ligands [141]. AUTOTACs bind the ZZ domain of the otherwise inactive autophagy receptor p62/Sequestosome-1/SQSTM1, which is activated into oligomeric bodies in complex with targets for their sequestration and degradation (Figure 13c). These chimeras were used to deplete a variety of oncoproteins and degradation-resistant aggregates in neurodegeneration at nanomolar concentrations in vitro and in vivo. AUTOTACs thus provide a direct tool to target not only the monomeric but also the oligomeric and aggregated species of the pathological hallmark proteins. There are several points to be fully investigated in future studies: (i) the off-target and selectivity features, (ii) how AUTOTACs can be recycled for multiple rounds of degradation, and (iii) whether they act catalytically and/or escape the lysosome.

Unlike AUTAC/ATTEC/AUTOTAC which utilize macroautophagy, CMA-based degraders harness chaperone-mediated autophagy (CMA) for protein degradation [142]. These molecules carry three functional groups: a cell membrane penetration sequence, a POI-binding sequence and a CMA-targeting motif (KFERQ) (Figure 13d). This strategy has been used to target numerous cytosolic proteins in neuronal cultures [143]. In a similar attempt, Xu and colleagues described that the polypeptide motif (MDFSGLSLIKLKKQ) on HIP1R (Huntingtin-interacting protein 1–related) possessed similar lysosomal targeting activity like the KFERQ motif and could be applied to lysosomal degradation of PD-L1 in cancer cells [144]. This discovery offers a novel path in immunotherapy. However, the low stability and delivery efficiency are among the obstacles for their druggability.

#### 2.2.2. Harnessing Endolysosomal Pathways

**Figure 14:**
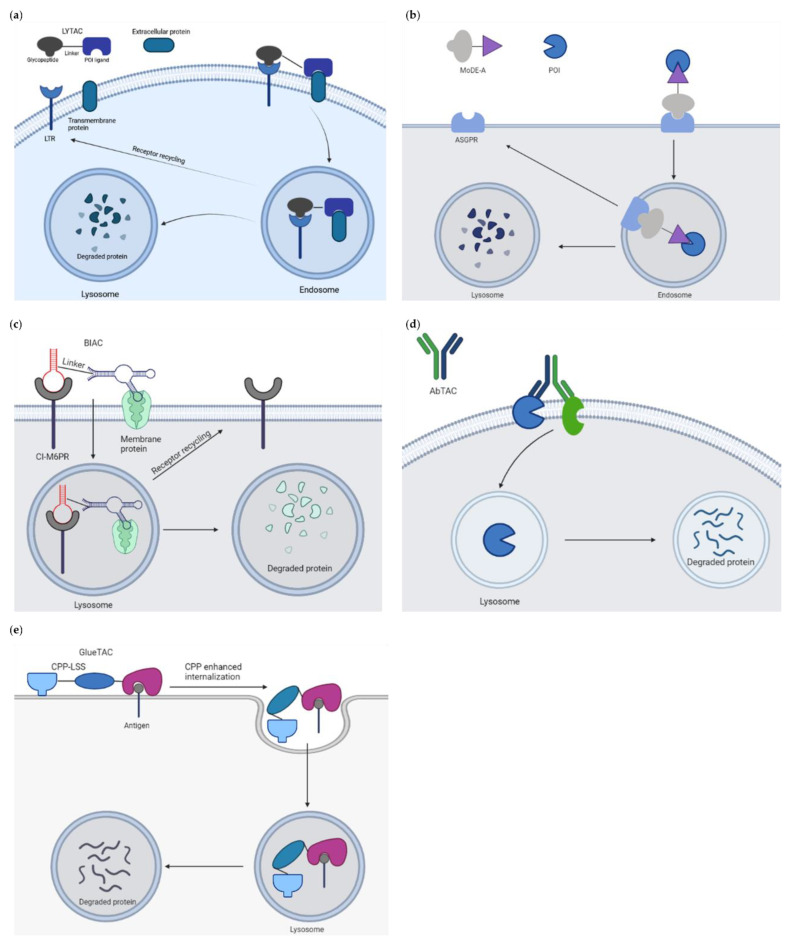
Concepts of degrader technologies hijacking lysosomal pathway. (**a**) LYTACs utilize a glycan tag to mark an extracellular POI for intracellular lysosomal degradation following shuttling receptor-mediated internalization. (**b**) Mechanism of action of MoDE-A bifunctional molecules. MoDE-A brings the target protein to ASGPR on hepatocytes for lysosomal degradation. (**c**) Bispecific aptamer chimeras use DNA aptamers to target CI-M6PR and transmembrane POI. (**d**) AbTAC recruits RNF43 to internalize cell surface proteins. (**e**) GlueTAC consists of a single-domain antibody, a cell-penetrating peptide and a lysosome-sorting sequence. The single-domain antibody is responsible for targeting POI and CPP-induced endocytosis of the complex followed by lysosomal degradation. ASGPR: asialoglycoprotein receptor; CI-M6PR: cation-independent mannose-6-phosphonate receptor; CPP-LSS: cell-penetrating peptide and lysosome-sorting sequence; LTR: lysosomal targeted receptor; POI: protein of interest; RNF43: RING finger protein 43.

The cytosolic localization of the UPS and targetable autophagy machinery restricts these approaches to POI with cytosolic domains and requires degraders to be cell permeable. To expand the scope of TPD to extracellular and membrane associated proteins that make up to 40% of all protein-encoding genes and involve growth factors, cytokines and other key agents in cancer and other diseases, a novel platform called LYTAC (LYsosome TArgeting Chimeras) has been developed (Figure 14a). These molecules bind and direct secreted and membrane proteins to lysosomes [145]. The first generation of LYTAC was composed of a target protein binder (a small molecule or an antibody) conjugated to a synthetic oligopeptide ligand, mannose-6-phosphonate (M6Pn). The first LYTACs used the cation-independent mannose-6-phosphonate receptor (CI-M6PR) as the endogenous lysosome-trafficking receptor. The M6Pn-LYTACs promoted internalization and lysosomal degradation of several therapeutically relevant proteins including apolipoprotein E4, epidermal growth factor receptor, CD71 and programmed death-ligand 1 by bridging them with CI-M6PR in HepG2 cells [145]. One drawback of this tool limiting its clinical applicability is that CI-M6PR is expressed in most tissues. To achieve a tissue-specific LYTAC activity, the second generation engaged the liver cell-specific asialoglycoprotein receptor (ASGPR) as the shuttling receptor [146]. An antibody or a small synthetic peptide was used as a binder linked to a triantenerrary N-acetylgalactosamine ligand that engaged ASGPR to drive the degradation of EGFR, HER2 and integrins, respectively, resulting in antiproliferative effects in hepatocellular carcinoma cells. These site-specific LYTACs improved pharmacokinetics in vivo. In a parallel study, Tang and colleagues similarly utilized ASGPR as the internalizing receptor for lysosomal induced degradation of several protein targets [147]. These reports hence establish LYTACs as a protein degradation modality with the ability to restrict degradation to a specific cell type expressing a given lysosome-targeting receptor.

In a similar attempt, Caianiello et al. developed a class of modular, bifunctional synthetic molecules termed MoDE-As (molecular degraders of extracellular proteins through the ASGPR) to drive the degradation of extracellular proteins [148]. MoDE-A molecules bridge POI to ASGPR on liver cells for endocytosis and lysosomal degradation (Figure 14b). MoDE-A induced depletion of both antibody and proinflammatory cytokine proteins in vitro and in vivo. MoDE-As possess several advantages compared to other TPD platforms: they are relatively small in size, monodispersed and nonprotein based. ASGPR is immunologically tolerogenic, reducing the likeliness of autoimmune responses to targeted proteins.

Recently, Han and colleagues constructed bispecific aptamer chimeras (BIAC) for lysosomal degradation of targeted membrane-associated proteins such as mesenchymal epithelial transition (Met) receptor and membrane receptor tyrosine protein kinase-like 7 (PTK-7), both known therapeutic cancer targets [149] (Figure 14c).

Wells and colleagues reported the development of an antibody-based PROTAC (AbTAC) that recruits membrane-bound E3 ligase RNF43 for the depletion of the cell-surface immune checkpoint protein programmed death-ligand 1 (PD-L1) [150] (Figure 14d). The event occurred in a lysosomal-dependent manner: incubation with bafilomycin, a lysosome acidification inhibitor, mitigated the degradation of PD-L1, whereas the proteasome inhibitor MG-132 did not. The lead AbTac AC-1 is a fully recombinant bispecific IgG, allowing not only for its rapid and renewable generation, but also for simple optimization of binding properties. It is built of human parts, limiting the chances to evoke an immune response. Thus, the technology holds the promise to expand the PROTAC field to target challenging membrane proteins. Another antibody-derived PROTAC strategy has been described by Zhang et al. [151] (Figure 14e). By conjugating with a cell-penetrating peptide and a lysosomal-sorting sequence, the resulting GlueTAC promoted the internalization and degradation of programmed death-ligand 1 (PD-L1) in vitro and in vivo, leading to sustained T cell activation and attenuated tumor growth in mice. This data provides an additional angle to degrade cell surface proteins.

#### 2.2.3. RIBOTACs

**Figure 15:**
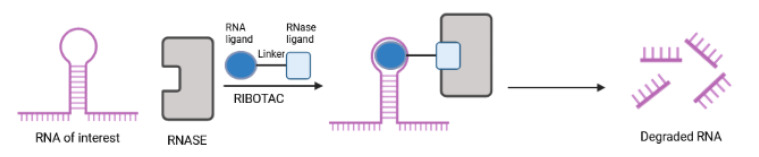
Schematic depiction of RIBOnuclease TArgeting Chimeras. The bivalent RIBOTAC molecules consist of an RNA-binding ligand (dark blue) and a ribonuclease (RNase) recruitment ligand (light blue) joined by a linker (black line). Upon binding a target RNA, RIBOTACs recruit an RNase in close proximity of the target, thereby promoting its destruction.

Another new strategy to combat cancer has been proposed through RIBOnuclease TArgeting Chimeras (RIBOTACs), which use RNA-targeting small molecules and RNase L, an otherwise latent ribonuclease, to accomplish the degradation of intracellular oncogenic RNAs (Figure 15). The first demonstration of a RIBOTAC as a potential cancer therapy involved selective cleavage of the miR-96 precursor in cancer cells in a catalytic and substoichiometric fashion [152]. Silenced miR-96 derepressed pro-apoptotic FOXO1 transcription factor, triggering apoptosis in breast cancer, but not in healthy breast cells [153]. Another small molecule called Targapremir-210 has been described to tackle triple negative breast cancer [154]. Here, silenced miR-210, an essential microRNA for cancer survival in hypoxic niches, derepressed glycerol-3-phosphate dehydrogenase 1-like enzyme (GPD1L), a hypoxia-associated protein, decreased HIF-1α, and triggered apoptotic cell death of diseased cells (MDA-MB-231) only under hypoxic conditions that are critical to the metastatic and invasive characteristics of cancer. In line with this, the module impaired the metastatic nature of these cells. Antiproliferative effects of Targapremir-210 could be reflected in a mouse xenograft model of hypoxic triple negative breast cancer. Compared to oligonucleotide-based/occupancy-based therapeutics, RIBOTACs offer many advantages [155]: (i) more favorable pharmacokinetic properties; (ii) catalytic nature (a RIBOTAC can recycle to another RNA); and (iii) low concentrations. Major drawbacks include challenging and time-consuming steps in design and development as well as slow cellular uptake due to high molecular weights [155]. Nevertheless, efforts to develop RIBOTACs for other disease-relevant RNAs that affect cellular responses to environmental conditions are anticipated in the near future.

## 3. Clinical Advances of Chemical Degraders in Oncology

**Figure 16:**
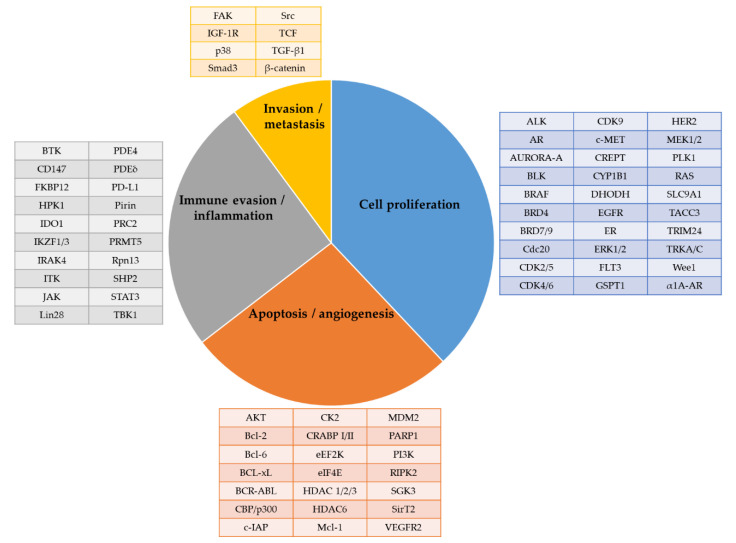
Summary of protein degradation target candidates involved in hallmarks of cancer. ALK: anaplastic lymphoma kinase; AR: androgen receptor; Bcl: B-cell lymphoma; BCL-xL: B-cell lymphoma-extra large; BRAF: v-raf murine sarcoma viral oncogene homolog B1; BRD: bromodomain-containing protein; BTK: Bruton’s tyrosine kinase; Cdc20: cell division cycle 20; CDK: cyclin-dependent kinase; cIAP: cellular inhibitor of apoptosis; CK2: casein kinase 2; CRABP I/II: cellular retinoic acid-binding protein 1/2; CREPT: cell cycle related and expression elevated protein in tumor; CYP1B1: cytochrome p450 family 1 subfamily B member 1; DHODH: dihydroorotate dehydrogenase; eEF2K: eukaryotic elongation factor 2 kinase; EGFR: epidermal growth factor receptor; eIF4E: eukaryotic initiating factor 4E; ER: estrogen receptor; ERK: extracellular signal-regulated kinase; FAK: focal adhesion kinase; FKBP: FK506-binding proteins; FLT3: fms-like tyrosine kinase 3; GSPT1: G1 to S phase transition 1; HDAC: histone deacetylase; HER: human epidermal growth factor receptor; HPK1: hematopoietic progenitor kinase 1; IDO1: indoleamine 2,3-dioxygenase 1; IGF-1R: insulin-like growth factor 1 receptor; IKZF: IKAROS zinc finger family; IRAK4: interleukin-1 receptor-associated kinase 4; ITK: interleukin-2-inducible T-cell kinase; JAK: janus kinase; Mcl-1: myeloid cell leukemia-1; MDM2: mouse double minute 2; MEK: mitogen-activated protein kinase kinase; PARP1: poly [ADP-ribose] polymerase 1; PDE: phosphodiesterase; PD-L1: programmed death-ligand 1; PI3K: phosphoinositide 3-kinases; PLK1: polo-like kinase 1; PRC: polycomb repressive complex; PRMT5: protein arginine methyltransferase 5; RAS: rat sarcoma; RIPK2: receptor interacting serine/threonine kinase 2; SGK3: serum/Glucocorticoid regulated kinase family member 3; SHP2: src homology 2 domain containing protein tyrosine phosphatase-2; SirT2: Sirtuin 2; SLC9A1: solute carrier family 9 member A1; STAT3: signal transducer and activator of transcription 3; TACC3: transforming acidic coiled-coil containing protein 3; TBK1: TANK-binding kinase 1; TCF: T-cell factor; TGF-β1: transforming growth factor beta 1; TRIM24: tripartite motif containing 24; TRKA/C: tropomyosin receptor kinase A/C; VEGFR: vascular endothelial growth factor receptor; α1A-AR: alpha-1A adrenergic receptor.

The first wave of protein degraders has focused on the oncology fields. The target proteins can be categorized into the following groups: (i) those involved in cancer cell proliferation; (ii) in apoptosis; (iii) in angiogenesis; (iv) in immune evasion or inflammation; and (v) in cancer invasion and metastasis (Figure 16) [50,51,156,157]. Among those, several have progressed into clinical activities for multiple disease indications (Table 3 and Table 4).

### 3.1. PROTAC-Based Clinical Trials

#### 3.1.1. AR PROTAC

The androgen receptor (AR) is a key driver of castration-resistant prostate cancer during the transition from a localized to a metastatic disease. Targeting the AR signaling axis with abiraterone, enzalutamide, darolutamide and apalutamide has demonstrated an overall survival benefit in the castration-sensitive state [158]. However, the response is short-lived; the disease remains invariably fatal. The majority of patients progressing on enzalutamide or abiraterone exhibit genetic alterations in the AR locus, either in the form of amplifications or point mutations in the AR gene [159,160]. Thus, destroying AR—and not simply inhibiting it—may change the treatment paradigm for this lethal disease.

ARV-110 (Bavdegalutamide) is the first-in-class PROTAC against AR. The treatment led to near-complete clearance of AR and suppressed AR-associated gene expression in vivo. ARV-110 inhibited the synthesis of prostate-specific antigen (PSA) and AR-dependent cancer cell proliferation by inducing apoptotic cell death. It also exhibited activity in enzalutamide refractory/resistant prostate cancer xenograft models with AR amplification and mutations with the exception of AR^L702H^ mutation and AR-V7 variance [159,160,161]. In phase I trials, patients with metastatic castration-resistant prostate cancer (mCRPC) who showed progression following at least two prior therapies (enzalutamide and/or abiraterone) received ARV-110 orally once or twice daily in sequential cohorts. Enhanced activity was observed in patients with specific molecular profiles including AR^T878^ and AR^H875^ mutations [162]. In phase II trials to assess antitumor activity, patients with mCRPC and ≥1 prior novel hormonal agent and/or chemotherapy were divided into 3 biomarker-specific groups: (i) AR^T878^ and/or AR^H875^ mutations; (ii) AR^L702H^ mutation or AR-V7; and (iii) wild-type AR or other AR alterations. A fourth subgroup involved patients based with a clinical history of less prior treatment strategy: ≤1 therapy for mCRPC, 1 novel hormonal agent, and no chemotherapy [162]. ARV-110 was applied at 420 mg once daily. The best PSA declines were achieved in patients with AR^T878A/S^ and/or AR^H875Y^ mutations. Of 7 RECIST (Response Evaluation Criteria in Solid Tumors)-evaluable patients with AR^T878A/S^ and/or AR^H875Y^ mutations, 6 exhibited tumor shrinkage. Among the most common treatment-related adverse events were nausea, fatigue, vomiting, decreased appetite, diarrhea and alopecia; none were grade ≥4 (NCT03888612). In a parallel study, the combination of ARV-110 with abiraterone will be assessed in patients with metastatic prostate cancer with PSA increase on abiraterone (NCT05177042). ARV-110 oral tablets in combination with abiraterone and a corticosteroid will be administered daily in 28-day cycles. ARV-766 is another oral AR PROTAC with a different profile than ARV-110 [163]. A phase I clinical trial is underway to evaluate the safety, tolerability, pharmacokinetics and pharmacodynamics in patients with mCRPC. The compound will be given once or twice daily in escalating doses (NCT05067140). Another phase I trial employs the oral PROTAC CC-94676 (AR-LDD) in patients with mCRPC who progressed on androgen deprivation therapy and at least one prior secondary hormonal therapy approved for CRPC (NCT04428788). CC-94676 displays similar preclinical activity as ARV-110 in regard to potent AR protein degradation, favorable pharmacokinetic properties, and sustained suppression of tumor growth in VCaP CRPC mouse models [161]. Yet it was not compared directly with ARV-110 for tumoricidal activity in a clinical setting. HP518, an oral PROTAC, has high activity of degrading wild-type AR and variant AR resistant to enzalutamide. An open-label study will evaluate its pharmacokinetics, safety and antitumor activity in patients with mCRPC. Participant are enrolled based on the following criteria: (i) metastatic disease at study entry proven by ≥2 bone lesions on bone scan or by soft tissue disease observed by CT/MRI; (ii) disease progression while receiving any androgen deprivation therapy, androgen biosynthesis inhibitors or second-generation AR inhibitors; (iii) recovery from toxicities related to any prior treatments; and (iv) ongoing androgen deprivation therapy with LHRH agonist/antagonist therapy or history of bilateral orchiectomy (NCT05252364). A further clinical trial in phase I is under way to evaluate the orally bioavailable AC176 in patients with mCRPC who have progressed on ≥2 prior systemic therapies (NCT05241613). In addition, a PROTAC degrader targeting AR-V7 splice variant and full-length AR is in development as a potential CRPC therapy.

#### 3.1.2. ER PROTAC

Around 80% of all newly diagnosed cases of breast cancer are estrogen receptor positive (ER^+^). While approved treatments have achieved great success in this patient population, many ER^+^ breast cancers develop resistance to therapy [164]. Fulvestrant, a selective estrogen receptor degrader (SERD), is the standard of care for ER^+^ metastatic breast cancer following anti-estrogen therapy. Even though fulvestrant has verified the importance of ER degradation as a therapeutic intervention, up to 50% of ER can remain when compared to baseline levels after six months of treatment. Several ER-directed PROTAC degraders are being tested in preclinical and/or clinical settings [165,166,167,168]. Unlike fulvestrant, which is administered via intramuscular injection, the ER-directed PROTAC ARV-471 is an oral therapy for women with ER^+^ metastatic breast cancer. In preclinical studies, ARV-471 robustly degraded ER in ER^+^ breast cancer cell lines, suppressed expression of ER-target genes (PR, GREB1, TFF) and inhibited cell proliferation of ER-dependent cell lines (MCF7, T47D) [167]. ARV-471 also affected clinically relevant ESR1 variants (Y537S and D538G) and suppressed growth of cell lines expressing those variants. ARV-471 showed in vivo activity in immature rat uterotrophic model, MCF7/E2 xenograft model, tamoxifen-resistant MCF7 mouse model, and ESR1 Y537S PDX model. ARV-471 exhibited improved in vivo activity compared to fulvestrant, which was further augmented by its combination with CDK4/6 inhibitors such as palbociclib.

Several clinical trials with ARV-471 are underway. A phase I, open-label study will recruit Japanese patients with ER^+^/HER2^-^ (human epidermal growth factor 2 negative) locally advanced or metastatic breast cancer to investigate the safety, tolerability and pharmacokinetics of ARV-471 (NCT05463952). The drug will be administered orally once daily with food in continuous dosing over 28-day cycles. A phase II neoadjuvant study will evaluate ARV-471 or anastrozole, a nonsteroidal aromatase inhibitor, in post-menopausal women with ER^+^/HER2^-^ localized breast cancer amenable to surgical resection to address the biological activity of respective compound (NCT05549505). ARV-471 will be administered once daily and 1 mg anastrozole will be given once daily until surgery, which will take place approximately 5.5 months after starting treatment. The safety, tolerability and clinical activity of ARV-471 alone or in combination with palbociclib are addressed in the ongoing dose escalation and cohort expansion study in patients with ER^+^/HER2^-^ advanced or metastatic breast cancer following prior chemotherapy or hormonal therapy (NCT04072952). The phase I part utilized traditional 3 + 3 dose escalation with ARV-471 oral tablets once daily for 28-day cycles in 21 heavily pre-treated patients with poorer prognosis. 48% of patients had visceral metastatic disease often in the liver and lung. All patients received previous CDK4/6 inhibitors; 71% of patients received fulvestrant; 38% of patients had chemotherapy; and 24% of patients had other selective ER degraders in the clinical trial setting. They had a median of 4 previous lines of therapy for their advanced or metastatic breast cancer. ARV-471 was well tolerated, the highest dose being 360 mg, and no grade 3/4 adverse events were observed. In paired tumor biopsy samples before and after treatment with ARV-471, mean ER degradation was significantly higher than with fulvestrant and has been observed in patients with either wild-type ER or mutant ER^Y537S^, ER^Y537N^, and ER^D538G^ in their tumors. A further study is recruiting to assess the combination of ARV-471 and everolimus, an mTOR inhibitor, in patients with advanced or metastatic ER^+^/HER2^-^ breast cancer who have received a prior CDK4/6 inhibitor and endocrine therapy in the advanced/metastatic setting (NCT05501769). Post-menopausal women or pre-/peri-menopausal women with ovarian suppression will receive ARV-471 oral tablets in combination with everolimus administered daily in 28-day cycles. A new sub-study aims to evaluate the safety and effects of ARV-471 when given together with the CDK4/6 inhibitor abemaciclib for the treatment of advanced or metastatic breast cancer (NCT05548127). The participants are enrolled based on the following criteria: (i) tumor is advanced or metastatic and cannot be fully treated by surgery or radiation therapy; (ii) tumor is sensitive to hormonal therapy; and (iii) tumor is nonresponsive to previous treatments. ARV-471 will be administered orally once daily, while abemaciclib will be given orally twice a day. The medication will continue until the tumors are not responding or side effects become too severe.

Other phase I clinical trials focus on AC682, an orally available ER degrader given as a single agent in 28-day cycles to address safety/tolerability, pharmacokinetics and antineoplastic activity in patients with ER^+^/HER2^−^ locally advanced or metastatic breast cancer (NCT05080842, NCT05489679). These studies either involve post-menopausal patients with life expectancy ≥3 months (NCT05080842) or those with ≥1 prior endocrine therapy regimen, concomitant use of CDK4/6 inhibitor being allowed (NCT05489679).

#### 3.1.3. BTK PROTAC

Bruton’s tyrosine kinase (BTK) plays a crucial role in B cell development, differentiation and signaling. BTK is closely associated with chronic B-cell receptor (BCR) activation, and is critical for the survival of B-cell neoplasms. Ibrutinib, a first-in-class covalent BTK inhibitor, has been approved for the treatment of several types of Non-Hodgkin’s lymphoma, specifically relapsed/refractory mantle cell lymphoma, chronic lymphocytic leukemia and Waldenström macroglobulinaemia. However, resistance eventually develops, highlighting the dire need for other strategies.

NX-2127 is not only a BTK PROTAC but also a molecular glue for IKZF1/3 degradation due to its cereblon binding activity to recruit ubiquitin ligase complex for BTK degradation [169]. The compound efficiently depleted not only wild-type (ibrutinib-sensitive) but also mutant BTK (ibrutinib-resistant BTK-C481S) with antitumor activity in vitro and in vivo. Major off-targets of ibrutinib including ITK, EGFR, and TEC were not affected upon PROTAC administration. The clinical safety and activity of NX-2127 are being evaluated in an ongoing phase 1a/1b study in adults with advanced B-cell malignancies who have received ≥ two prior lines of therapy (or one for patients with Waldenström macroglobulinaemia) and for whom no other therapies are known for clinical benefit (NCT04830137). A patient with R/R DLBCL (who had 4 prior lines of therapy) receiving 300 mg daily of NX-2128 experienced a complete response at 8 weeks which was confirmed at 16 weeks and remains ongoing [170]. The complete response included dramatic reductions in lymph node size and resolution of abnormal metabolic activity to background levels. The clinical response was assigned to degradation of BTK and IKZF1/3. In a similar vein, a dose escalation and cohort expansion study is recruiting participants to assess the safety and preliminary efficacy of the oral BTK PROTAC NX-5948 for treatment of advanced B-cell malignancies (NCT05131022). Further dose escalation and expansion trials of the oral degrader BGB-16673 are underway in patients with B-cell malignancies (NCT05006716, NCT05294731). HSK29116, another oral degrader, is currently being evaluated in a phase 1a/1b multi-center study to assess its safety and antineoplastic activity in patients with relapsed/refractory B-cell malignancies (NCT04861779).

#### 3.1.4. BRD4 PROTAC

BRD4, a member of the bromo- and extra-terminal (BET) family of proteins, recruits transcriptional regulatory complexes to acetylated chromatin, thereby controlling specific networks of genes critical for proliferation and cell cycle progression [171]. Alterations in regulation of BRD4 activities have been allied with cancer and inflammatory diseases. The catalytic role of BRD proteins in transcription led to the development of small-molecule inhibitors against BRDs. JQ1, the first BRD inhibitor reported [172], marks a success story of BRD4 as a novel therapeutic vulnerability, and paved the way for several BRD inhibitors with some being under clinical evaluation [171,173,174,175,176,177,178,179]. However, early phase clinical trials show only modest clinical activity as single agents in patients with advanced cancer [171,179]. This can be explained by the assumption that small molecule BRD inhibitors may only block their chromatin binding function but spare other functional domains [171]. This highlights the unmet need for more effective strategies to treat diseases with BRDs as their Achilles’ heel. To date several structurally different BRD4-directed PROTAC degraders have been described [109,180,181,182,183,184,185,186,187,188,189,190,191,192]. RNK05047 is a BRD4 degrader in a phase I/II study in patients with advanced solid tumors including diffuse large B-cell lymphoma (NCT05487170) [184]. The compound will be administered intravenously to assess its safety, tolerability, pharmacokinetics, pharmacodynamics and clinical activity.

#### 3.1.5. BRD9 PROTAC

Bromodomain-containing protein 9 (BRD9), a specific component of the non-canonical mammalian SWI/SNF chromatin remodelling complex, is essential to maintain the transformed phenotype of acute myeloid leukemia and the growth of synovial sarcoma [161,193]. This makes BRD9 protein an appealing drug target. However, BRD9 has been considered undruggable using currently available modalities: patients with advanced synovial sarcoma benefit from very limited therapeutic options with median overall survival of only 18 months. CFT8634 is an orally bioavailable degrader against BRD9 for the treatment of synovial sarcoma and SMARCB1-deleted solid malignancies [194]. It is superior to existing BRD inhibitors due to high specificity towards BRD9 over BRD4/7 and improved toxicity profile. CFT8634 demonstrated potent antitumor activity in both cell line and patient-derived xenograft models of synovial sarcoma when administered orally [194]. The safety, tolerability, pharmacokinetics, pharmacodynamics, and tumoricidal activity of CFT8634 are assessed in an ongoing phase 1/2 study in patients with locally advanced or metastatic SMARCB1-perturbed cancers, including synovial sarcoma and SMARCB1-null tumors with unresectable or metastatic disease, who received ≥1 prior line of standard of care systemic therapy and for whom no other therapies are known to confer clinical benefit (NCT05355753). FHD-609 is another BRD9 PROTAC that is currently under clinical evaluation in a phase I dose escalation and expansion study in patients with advanced synovial sarcoma (NCT04965753). Unlike the oral CFT8634, FHD-609 is given intravenously every 2 weeks. Initial clinical data from 2 patients showed degradation of BRD9 in on-treatment metastatic tumor biopsies.

#### 3.1.6. STAT3 PROTAC

STAT3, a protein that has been historically undruggable, is a transcriptional regulator allied with numerous cancers and other inflammatory and autoimmune disorders. Under steady state conditions, the STAT3 protein is activated by cytokines and growth factors, thereby triggering transcriptional regulation of many cellular functions, while under stress conditions such as cancer, STAT3 activity becomes dysregulated, resulting in persistent activation of STAT3, which is associated with poor prognosis. Selective degraders targeting STAT3 thus have the potential to provide an intriguing solution to address multiple STAT3-dependent pathologies. KT-333 is a first-in-class PROTAC of STAT3. The FDA has granted orphan drug designation to KT-333 for the treatment of peripheral T-cell lymphoma, a disease entity with dysregulated STAT3 for which no approved therapies are available [195]. An open-label study enrolls adults with refractory lymphoma, large granular lymphocytic leukemia and solid tumors to evaluate the safety, tolerability, pharmacokinetics and pharmacodynamics of KT-333 when administered intravenously weekly in 28-day cycles (NCT05225584).

#### 3.1.7. BCL-xL PROTAC

B-cell lymphoma extra large (BCL-xL) is a well-validated cancer target. However, the on-target and dose-limiting thrombocytopenia limits the use of BCL-X_L_ inhibitors, such as orally bioavailable ABT263, as safe and effective anticancer agents. DT2216 is a selective BCL-xL degrader utilizing ABT263 and a VHL E3 ligase binder [196]. DT2216 demonstrated superior potency against various BCL-xL-dependent leukemia and cancer cells but considerably less toxicity to platelets than ABT263 in vitro due to the lack of significant VHL expression in platelets. Anticancer activity was verified in several xenograft tumors as a single agent (MOLT-4 T-ALL xenograft) or in combination with other therapeutic agents (H146 SCLC xenograft, MDA-MB231 breast cancer xenograft, and T-ALL PDX models), without causing significant thrombocytopenia. DT2216 selectively killed various BCL-xL-dependent T-cell lymphomas (TCL) cells including MyLa cells in vitro [197]. DT2216 alone was highly effective against MyLa TCL xenografts in mice without causing appreciable thrombocytopenia or other toxicity. Furthermore, DT2216 combined with ABT199, a selective Bcl-2 inhibitor, synergistically reduced disease burden and improved survival in a TCL PDX mouse model dependent on both Bcl-2 and BCL-xL. A recent study reported that BCL-xL is highly expressed within the tumor-infiltrating (TI)-Treg population from renal cell carcinoma and several other human cancers. Proteasomal degradation of BCL-xL using two independent PROTACs (DT2166 and PZ15227 with a CRBN E3 ligase ligand) induced apoptosis of TI-Tregs and the activation of TI-CD8^+^ T cells [198]. These activities resulted in a potent suppression of syngeneic tumor growth with no detectable damage within several normal tissues or thrombocytopenia in immunocompetent, but not in immunodeficient or CD8^+^ T cell-depleted mice. Thus, BCL-xL destruction represents a novel avenue for cancer immunotherapy. Based on these promising preclinical findings, DT2216 is under clinical evaluation in a dose escalation and cohort expansion study for its safety, tolerability, and activity in patients with relapse and refractory solid tumors and hematologic malignancies when administered intravenously in 28-day cycles (NCT04886622).

#### 3.1.8. Other PROTACs

CFT1946 is an orally bioavailable mutant selective degrader of BRAF^V600X^, which accounts for approximately 50,000 cancer diagnoses annually, to offer more durable responses. The bifunctional CFT1946 received “the study may proceed letter” from the FDA to initiate a phase I/II clinical study in patients with BRAF^V600^ mutant solid cancers including lung, colorectal and melanoma [199]. The trial initiation is expected by the end of 2022. CFT1946 is active in vitro and in vivo in models with BRAF^V600E^-driven disease and in the escape mutant BRAF^V600E^/NRAS^Q61K^-driven model [200]. In preclinical settings, BRAF^V600E^ degradation by CFT1946 caused loss of MAPK signaling superior to inhibition alone and attenuated viability of BRAF^V600E^ cells but not wild-type BRAF cells. Another PROTAC which has been approved for the investigational new drug (IND) application is the world’s first-in-class NTRK degrader (neurotrophic tyrosine receptor kinase; CG001419) for the treatment of advanced solid tumors [201]. One PROTAC that is in late preclinical development and planned for an IND application submission is CFT8919, an orally bioavailable mutant selective degrader of EGFR^L858R^ in non-small cell lung cancer [202]. Preclinical data show that CFT8919 induced tumor regression in mouse models resistant to first and third generation EGFR inhibitors. CFT8919 also exhibited intracranial activity, reducing tumor burden in a brain metastasis model. This indicates its potential to be active in the central nervous system. A further molecule planned to be filed for an IND application in late 2022 is KT-253, a first-in-class MDM2 degrader. Degradation of MDM2, rather than inhibition, has the ability to block the feedback loop which upregulates MDM2 production and effectively drives MDM2-dependent tumor cells to rapid apoptosis by robust p53 stabilization [203]. KT-253 exhibited improved potency relative to reversible small-molecule inhibitors leading to potent in vitro and in vivo efficacy that is superior to all clinically active agents. Sustained tumor regression was achieved in vivo in leukemia models following just a single dose [203]. As wild-type p53 is present in >50% of tumors, KT-253 represents another program with broad potential in liquid and solid tumors.

### 3.2. Molecular Glue-Based Clinical Trials

IMiDs are among the standard of care for treatment of multiple myeloma and have shown activity in some non-Hodgkin’s lymphoma subtypes. Given that many patients treated with these agents often develop disease progression, an unmet need remains. New generation IMiDs have been designed for better clinical efficacy [121]. Avadomide (CC-122) displays antitumor properties and modulation of immune cells [119,204]. It binds CRBN to degrade IKZF1 and IKZF3, triggering apoptosis of DLBCL cell lines and blocking tumor growth in xenograft mouse models. Avadomide is in clinical trials for non-Hodgkin’s lymphoma, DLBCL, hepatocellular carcinoma, B-cell chronic lymphocytic leukemia, multiple myeloma, chronic lymphocytic leukemia/small lymphocytic lymphoma, follicular lymphoma, primary liver cancer, advanced solid tumors and melanoma as a single agent or in combination with other agents (NCT03283202, NCT03834623, NCT03310619, NCT02509039, NCT02859324, NCT02417285, NCT02406742, NCT02323906, NCT01421524, NCT02031419). Iberdomide (CC-220) induces CRBN catalyzed proteasomal degradation of IKZF1 and IKFZ3 in monocytes, B and T cells. The drug is under clinical evaluation as monotherapy or in combination with other agents for the treatment of newly diagnosed multiple myeloma, relapsed or refractory multiple myeloma, (smoldering) plasma cell myeloma and relapsed or refractory lymphoma (NCT05560399, NCT04998786, NCT05354557, NCT04564703, NCT05177536, NCT04392037, NCT05392946, NCT05527340, NCT04776395, NCT05169515, NCT04975997, NCT05434689, NCT05199311, NCT05289492, NCT04884035, NCT04855136, NCT05272826, NCT04464798, NCT05558319, NCT03310619, NCT04934475, NCT02773030). CC-99282 is a novel, oral IKZF1/3 targeting CRBN E3 ligase modulator agent with an improved substrate degradation profile compared with other IKZF1/3 degrading agents such as lenalidomide, avadomide, and iberdomide. CC-99282 showed enhanced antiproliferative, apoptotic and immune-stimulatory activity in a range of follicular lymphoma and DLBCL models, including those with chemoresistance [205,206]. It displayed a robust distribution profile that favors target tissues such as lymphoid organs. CC-99282 acted synergistically in combination with anti-CD20 monoclonal antibody rituximab treatment [206]. Tumoricidal activity was validated in vivo, resulting in improved tumor regression and tumor-free animals in several lymphoma xenograft models, including an intracranial xenograft model [206]. A recent analysis in >20 DLBCL cell lines showed that loss of IKZF1/3 is necessary but not sufficient for CC-99282 efficacy [207]. An explanation has been provided by the observation that IKZF1 regulated gene expression via histone modifications, including polycomb repressive complex PRC2-mediated histone H3 lysine 27 trimethylation [207]. Consistently, pathway analysis upon exposure to CC-99282 or tazemetostat, an EZH2 inhibitor, demonstrated high overlap between pathways altered by both agents. Combination treatment led to additive and/or synergistic antiproliferative and apoptotic effects in DLBCL cell lines. No alterations were observed in IKZF1 degradation or overall H3K27me3 status but downstream effects were enhanced. Combination treatment yielded durable responses and tumor-free animals [207]. CC-99282 is currently under investigation in phase I clinical studies in patients with relapsed/refractory non-Hodgkin’s lymphomas and chronic lymphocytic leukemia/small lymphocytic lymphoma as monotherapy or in combination with other agents (NCT04434196, NCT04884035, NCT03930953, NCT05169515, NCT03310619). CFT7455 is another orally available next-generation IKZF1/3 degrader for the treatment of hematological malignancies. Rapid and deep degradation of these targets from malignant B cells resulted in tumor cell death, and depletion from the tumor microenvironment resulted in T-cell activation [208,209]. In in vitro and in vivo models of multiple myeloma and non-Hodgkin’s lymphoma, including anaplastic large cell lymphoma, diffuse large B-cell lymphoma, and mantle cell lymphoma, CFT7455 demonstrated higher binding affinity to CRBN E3 ligase complex and greater activity than other investigational and approved agents in similar classes [208,209]. A first-in-human, phase I dose escalation and expansion study is currently enrolling patients with relapsed/refractory non-Hodgkin’s lymphoma or multiple myeloma (NCT04756726). The purpose of the trial is to characterize the safety, tolerability, pharmacokinetics, pharmacodynamics, and antitumor activity of CFT7455 administered orally according to different dosing schedules as a single agent and in combination with dexamethasone. Exploratory objectives include characterization of target engagement and IKZF1/3 degradation and assessing the immunomodulatory effects of CFT7455. CC-92480 is another oral CRBN E3 ligase modulator currently under clinical investigation in patients with newly diagnosed and/or refractory/relapsed multiple myeloma (NCT03374085, NCT05552976, NCT05519085, NCT03989414, NCT05372354). CC-92480 induces rapid degradation of the transcription factors IKZF1/3, leading to immune-stimulatory effects and apoptosis of myeloma cells, including those resistant to lenalidomide and pomalidomide [210,211,212,213]. CC-92480 in combination with dexamethasone and the proteasome inhibitor bortezomib appears to be safe and well tolerated with encouraging preliminary efficacy in patients with refractory/relapsed multiple myeloma. The overall response rate across all doses per investigator assessment was 73.7% (14/19 pts), including 3 stringent complete responses and 1 complete response [211]. Parallel trials are ongoing to determine the recommended phase II dose of CC-92480 in combination with standard treatments including carfilzomib, daratumumab, elotuzumab, isatuximab or with novel inhibitors tazemetostat, BMS-986158 and trametinib.

Interleukin-1 Receptor-Associated Kinase 4 (IRAK4) is a key mediator of innate immunity. It is found hyperactivated in a variety of autoimmune diseases. Multiple small-molecule kinase inhibitors of IRAK4 have been developed to block its kinase activity in autoimmune, inflammatory and oncological diseases, with the most advanced being in phase II clinical trials. However, some reports have indicated a nonkinase function for IRAK4 in several cell types, pointing out the need of alternative therapeutic interventions [214]. KT-413 is a novel IRAKIMiD degrader of IRAK4 and IMiD substrates. An early trial is under way to evaluate the safety, tolerability, pharmacokinetics and pharmacodynamics of KT-413 in patients with MYD88 mutant and MYD88 wild-type relapsed or refractory DLBCL when administered intravenously (NCT05233033). The trial will also explore target (IRAK4/IKZF1/IKZF3) knockdown and downstream effects in peripheral blood mononuclear cells and tumors.

Malignant tumors must evade the immune system for continuous growing, a hurdle some malignancies may overcome by attracting immune-suppressive regulatory T cells (Tregs). The zinc finger transcription factor IKZF2 (Helios) is critical for the activity and stability of Tregs; its deficiency enhances immune responses to tumors in vivo, indicating IKZF2 may be an appealing target for cancer immunotherapy. DKY709 is the first CRBN modulator agent targeting IKZF2/4 with no activity towards IKZF1/3. Upon exposure to DKY709, human Tregs showed reduced suppressive activity [215]. Oral treatment with DKY709 drove a rapid and sustained degradation of IKZF2 including in humans and led to delayed tumor growth in mice with humanized immune systems and enhanced immunization responses in monkeys [215]. DKY709 is currently being investigated in a phase I trial in patients with advanced solid tumors as an immune-enhancing agent for cancer immunotherapy alone or in combination with other immunotherapy agents (NCT03891953).

Other CRBN modulators including CC-885, CC-90009, ZXH-1-161 and oral bioavailable candidates SJ6986 and MRT-2359 exhibit therapeutic potential for GSPT1 degradation [216,217,218,219,220]. CC-90009 is being tested in clinical trials for the treatment of patients with acute myeloid leukemia or myelodysplastic syndrome (NCT02848001 and NCT04336982), while a phase I/II clinical trial testing MRT-2359 in MYC-driven tumors is under way (NCT05546268).

## 4. Conclusions

Over the last two decades, the field of targeted protein degradation has evolved successfully, but almost all of this progress has been catalyzed through PROTACs alone. PROTACs have demonstrated excellent preclinical activities in multiple types of cancers by robust and selective depletion of their targets. Potent tumoricidal activities could also be validated in some in vivo models that were resistant to the counterpart small-molecule inhibitors of PROTACs. Despite their success, there are still some challenges concerning optimal druggability and evaluation of biological activity. Artificial intelligence and virtual drug screening platforms may improve identification of highly specific POI/E3 ligands. Molecular dynamic simulations, X-Ray crystallography and cryo-electron microscopy may be effective techniques for determining better ternary complex structures of PROTAC with POI and E3 ligase [221]. Drug-like properties may be improved by cell permeable precursors or by computer aided drug design softwares [51,55]. Clinical delivery and metabolic stability may be advanced by application of prodrug PROTACs, antibody-drug conjugates, folate-PROTACs, aptamer-PROTAC conjugates and nanoparticle based PROTACs to penetrate deep into tumors [56,65,66,67,68,109,222].

**Figure 17:**
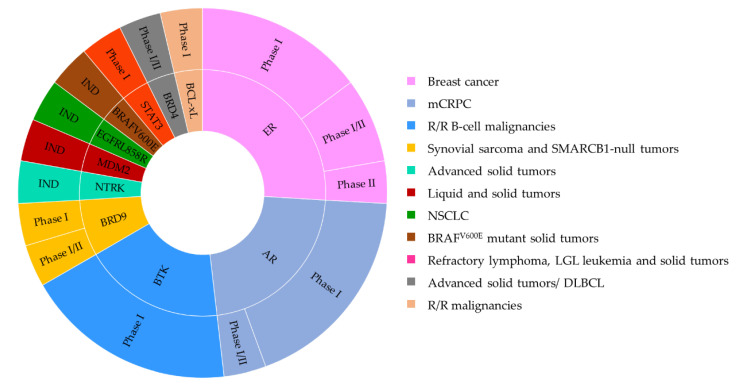
Overview of PROTAC degraders in clinical trials for cancer treatment. AR: androgen receptor; BCL-xL: B-cell lymphoma-extra large; BRAF: v-raf murine sarcoma viral oncogene homolog B1; BRD: bromodomain-containing protein; BTK: Bruton’s tyrosine kinase; DLBCL: diffuse large B cell lymphoma; EGFR: epidermal growth factor receptor; ER: estrogen receptor; IND: investigational new drug, LGL: large granular lymphocyte; mCRPC: metastatic castration-resistant prostate cancer; MDM2: mouse double minute 2; NSCLC: non-small cell lung cancer; NTRK: neurotrophic tyrosine receptor kinase; R/R: relapsed/refractory; SMARCB1: SWI/SNF related, matrix associated, actin dependent regulator of chromatin, subfamily B, member 1; STAT3: signal transducer and activator of transcription 3.

**Figure 18:**
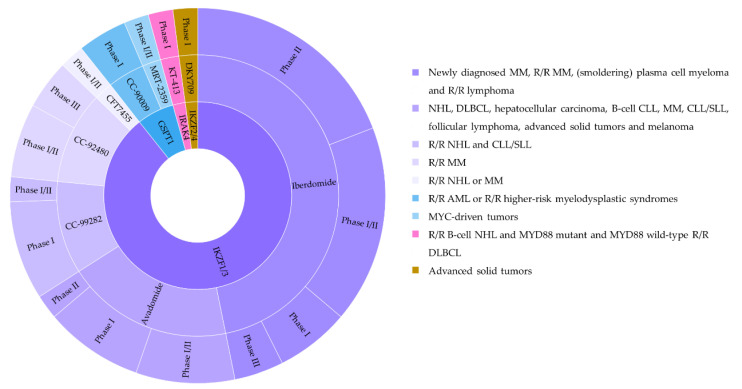
Overview of molecular glues in clinical trials for cancer treatment. AML: acute myeloid leukemia; CLL: chronic lymphocytic leukemia; DLBCL: diffuse large B cell lymphoma; IKZF: IKAROS zinc finger family; IRAK4: interleukin-1-receptor-associated kinase 4; GSPT1: G_1_ to S phase transition 1; MM: multiple myeloma; MyD88: myeloid differentiation primary response 88; NHL: non-Hodgkin’s lymphoma; R/R: relapsed/refractory; SLL: small lymphocytic lymphoma.

Targeted protein degradation is one of the fastest growing fields. Within a relatively short time, the platform is already impacting medicine as multiple therapeutic degraders have entered the stage of practical application from conceptual frameworks (Figure 17 and Figure 18). Clinical PROTAC degraders of AR and ER with minor off-target toxicities harbor favorable pharmacokinetic profiles and are well-tolerated by heavily pretreated patients with advanced/refractory prostate or ER^+^/HER2^-^ breast cancer, respectively. Although degrader drugs that are under clinical investigation (PROTAC and molecular glues, see Table 3 and Table 4, Figure 17 and Figure 18) or already on the market (IMiDs) attack a variety of cancers, other disease indications may also benefit from this emerging drug paradigm (Table 5). A topical AR-PROTAC compound (GT20029) in phase I clinical trials could treat patients with androgenetic alopecia and acne (NCT05428449). An orally bioavailable heterobifunctional IRAK4 degrader (KT-474) in phase I clinical trials (NCT04772885) could treat patients with various immuno-inflammatory diseases, such as rheumatoid arthritis, atopic dermatitis or hidradenitis suppurativa. A pilot study suggested a favorable benefit/risk ratio in systemic lupus erythematosus for iberdomide, a drug with an immunomodulatory mechanism of action, supporting further clinical investigation (NCT02185040 and NCT03161483) [223]. Other clinical studies explore whether iberdomide and its analog avadomide are safe and effective in patients with severe renal impairment (NCT04933747, NCT03097016). With the discontinuation of the phase III trial of tominersen, a huntingtin protein (HTT)-silencing antisense oligonucleotide therapy, targeted degrader modalities could become the first disease-modifying therapy for the neurodegenerative Huntington disease: two TPD strategies have already been reported to reduce mHTT levels [139,224]. Targeted degraders could also emerge as potential antiviral agents: a RIBOTAC molecule binds and degrades a functional structure within the SARS-CoV-2′s RNA genome, the causative agent of the global pandemic COVID-19, thereby triggering the inhibition of viral propagation [225].

Results of ongoing clinical trials are expected to establish the clinical significance of degrader drugs in oncology and other disease entities. Evidence of long-term effects or potent toxicity is still missing. Future clinical investigation will put more emphasis on the assessment of the clinical efficacy and adverse events in larger cohorts, exploration of potential resistance mechanisms, defining biomarkers for optimal selection of patients and exploration of potential synergy with other cancer therapeutics. In fact, early clinical investigations suggest that combination of chemical degraders either with targeted inhibitors or with chemotherapy/other agents may represent a robust alternative path for cancer therapy: ARV-471 produced a synergistic tumoricidal effect when combined with the CDK4/6 kinase inhibitor.

Potentials of targeted degradation applications go well beyond what was thought to be possible. PROTACs could be used as immune-enhancing agents. In combination with immune checkpoint inhibitors, they could augment the ability of the immune system to recognize and erase tumors. Degrader systems against immune checkpoint proteins and those that modulate T-cell receptor function have already been developed [144,150,151,221,226,227]. These may show improved pharmacokinetic and toxicity profiles when compared to antibody-based inhibitors. Two degrader agents are under clinical investigation as a novel avenue for cancer immunotherapy (NCT04886622 and NCT03891953). A recent study described the first tumor microenvironment-reprogramming nanoPROTAC strategy synergized with phototherapy that specifically revived antitumor immunity [228]. Such molecules in combination with other cancer treatments (e.g., chemo-/targeted/radiotherapy) could be utilized to intervene with immune-associated signaling pathways including hypoxia, lipid metabolism, glycolysis, glutaminolysis and adenosine signaling.

The incorporation of new elements into PROTAC technologies may help taming the undruggable drug targets and thus broaden the landscape of targeted degradation. This is highlighted by recent reports of utilizing RNA and DNA as respective recruiting elements to eliminate unwanted targets for cancer prevention and treatment [92,96,99,100,101]. In addition, a range of additional TPD approaches independent of the proteasomal pathway fuels the expansion of the drug target spectrum, although further investigations are warranted [137,138,139,140,141,145,146,147,148]. Thus, targeted degradation holds enormous promise to deliver medicine to the clinic in ways that are impossible with other modalities. We believe that the field of TPD will continue progressing with its rapid pace to maximize the benefits for patients and change the course of many deadly disease entities.

## Figures and Tables

**Table 1:** Characteristics of protein suppressive strategies. PO: per oral; IV: intravenous injection; SC: subcutaneous injection.

	Main Approaches To Suppress Target Of Interest			
	PROTAC	Monoclonal Antibody	Small Molecule Inhibitor	Gene-Editing Via Nucleic Acids
**Selectivity**	+++	++	+	++
**Route Of Administration**	PO/IV/SC	IV/SC	PO/IV/SC	IV/SC
**Target Of Interest**	Protein on cell surface and inside a cell	Protein on cell surface	Protein on cell surface and inside a cell	DNA or RNA
**Metabolic Stability**	++	-/+	++	-/+
**Tissue/Cell Penetration**	+	-/+	++	-/+
**Concentrations Required**	Substoichiometric	N/A	Stoichiometric	N/A
**Active Binding Site**	- (Can target undruggable and mutated proteins)	+++ (Can target undruggable proteins)	+++ (Cannot target mutated proteins)	- (Can target undruggable proteins)
**Inhibitory Outcome**	Blockade of both enzymatic and scaffolding functions	N/A	Impaired enzymatic function	N/A
**Elimination Of POI**	+++	-	-	+++
**Catalytic Mechanism Of Action**	+++	-	-	+++
**Systemic Delivery**	+++	+++	+++	-

**Table 2:** Comparison of different degrader systems. AbTac: antibody-based PROTAC; ATTEC: autophagy tethering compound; AUTAC: Autophagy Targeting Chimera; AUTOTAC: AUTOphagy TArgeting Chimera; BIAC: bispecific aptamer chimera; CMA: chaperone-mediated autophagy; GlueTac; GlueBody Chimera; LYTAC: LYsosome TArgeting Chimera; Mode-A; molecular degraders of extracellular proteins through the ASGPR; PROTAC: PROteolysis TArgeting Chimera; RIBOTAC: RIBOnuclease TArgeting Chimera; RNA: ribonucleic acid; SNIPER: Specific and non-genetic inhibitors of apoptosis protein (IAP)-dependent protein eraser.

Degradation Pathways	Degradation Systems	Degradable Targets	Advantages	Limitations	Highest Phase
**Ubiquitin-proteasome**	PROTAC	Intracellular proteins	In vivo, oral, improved selectivity and efficiency, does not require tight binding, catalytic and substoichiometric, definite structure and mechanism	High molecular weight, high surface area	Phase II
	Molecular glue	Intracellular proteins	Good pharmacology, specific to ligase and substrate	Mainly relies on accidential discovery	Approved
	SNIPER	Intracellular proteins	Catalytic and substoichiometric, definite structure and mechanism	Dependent on E3 ligase IAPs	Exploratory
**Autophagy**	AUTAC	Cytoplasmic proteins, fragmented organelle	Broader targets	Lack of detailed mechanism for K63 ubiquitination	Exploratory
	ATTEC	Intracellular proteins, nonproteins	Broader targets, low molecular weight, good transmembrane activity, better pharmacokinetics	Lack of detailed interaction between LC3 and degraders, high molecular design costs, low versatility	Exploratory
	AUTOTAC	Degradation-resistant aggregates	Broader targets	Unclarified mechanism	Exploratory
	CMA-based	Transmembrane proteins, aggregates	High specificity	Low stability and delivery efficiency, dependent on cell penetrating peptides, limited therapeutic effects	Exploratory
**Lysosomal**	LYTAC	Extracellular proteins, membrane-associated proteins	Broader targets	Limited clinical applicability, required an antibody or a small synthetic peptide to maintain its characteristics, difficult to determine the optimal linking site, high molecular weight, induced immunogenicity	Exploratory
	MoDE-A	Extracellular proteins	Small in size, monodisperse and nonprotein based, well-tolerated immunogenicity	Relies on highly expressed ASGPR on hepatocytes	Exploratory
	BIAC	Membrane-associated proteins	Costless and easily synthesized	In the early stages	Exploratory
	AbTAC	Transmembrane proteins	Utilizes two specific binding sites of bispecific antibodies to recruit E3, simple optimization of binding properties, built of human parts, reduced immune response	High molecular weight	Exploratory
	GlueTAC	Cell surface proteins	Cell-type independent degradation strategy, high specificity and efficiency	In the early stages	Exploratory
**Ribonuclease**	RIBOTAC	RNA	Favorable pharmacokinetic profile, low concentration, catalytic	High molecular weight, slow cellular uptake	Exploratory

**Table 3:** Summary of PROTACs under clinical evaluation for cancer therapy. Yellow: recruiting; violet: not yet recruiting. AR: androgen receptor; BCL-xL: B-cell lymphoma-extra large; BRAF: v-raf murine sarcoma viral oncogene homolog B1; BRD: bromodomain-containing protein; BTK: Bruton’s tyrosine kinase; DLBCL: diffuse large B cell lymphoma; EGFR: epidermal growth factor receptor; ER: estrogen receptor; IND: investigational new drug; LGL: large granular lymphocyte; mCRPC: metastatic castration-resistant prostate cancer; MDM2: mouse double minute 2; NSCLC: non-small cell lung cancer; R/R: relapsed/refractory; STAT3: signal transducer and activator of transcription 3; NTRK: neurotrophic tyrosine receptor kinase; 2H2022: Second half of the year 2022.

Time	Degrader	Target	Indication	NCT Number	Phase
2019 2022	ARV-110	AR	mCRPC	NCT03888612 NCT05177042	Phase I/II Phase I
2021	ARV-766	AR	mCRPC	NCT05067140	Phase I
2020	CC-94676	AR	mCRPC	NCT04428788	Phase I
2022	HP518	AR	mCRPC	NCT05252364	Phase I
2022	AC176	AR	mCRPC	NCT05241613	Phase I
2021	DT2216	BCL-xL	R/R malignancies	NCT04886622	Phase I
2022	RNK05047	BRD4	Advanced solid tumors/ DLBCL	NCT05487170	Phase I/II
2022	CFT8634	BRD9	Synovial sarcoma and SMARCB1-null tumors	NCT05355753	Phase I/II
2021	FHD-609	BRD9	Advanced synovial sarcoma or advanced SMARCB1-null tumors	NCT04965753	Phase I
2021	NX-2127	BTK	R/R B-cell malignancies	NCT04830137	Phase I
2021	NX-5948	BTK	R/R B-cell malignancies	NCT05131022	Phase I
2021 2022	BGB-16673	BTK	B-cell malignancies	NCT05006716 NCT05294731	Phase I Phase I
2021	HSK29116	BTK	R/R B-cell malignancies	NCT04861779	Phase I
2019 2022 2022 2022 2022	ARV-471	ER	ER^+^/HER2^-^ locally advanced or metastatic breast cancer	NCT04072952 NCT05501769 NCT05463952 NCT05549505 NCT05548127	Phase I/II Phase I Phase I Phase II Phase I/II
2021 2022	AC682	ER	ER^+^/HER2^-^ locally advanced or metastatic breast cancer	NCT05080842 NCT05489679	Phase I Phase I
2022	KT-333	STAT3	Refractory lymphoma, LGL leukemia and solid tumors	NCT05225584	Phase I
2022	CFT1946	BRAF^V600E^	BRAF-V600E mutant solid tumors		IND
Planned for (2H2022)	CFT8919	EGFR^L858R^	NSCLC		IND
Planned for (2H2022)	KT-253	MDM2	Liquid and solid tumors		IND
2022	CG001419	NTRK	Advanced solid tumors		IND

**Table 4:** Summary of molecular glues under clinical evaluation for cancer therapy. Red: active, not recruiting; yellow: recruiting; blue: completed; violet: not yet recruiting. AML: acute myeloid leukemia; CLL: chronic lymphocytic leukemia; DLBCL: diffuse large B cell lymphoma; GSPT1: G_1_ to S phase transition 1; IKZF: IKAROS zinc finger family; IRAK4: interleukin-1 receptor-associated kinase 4; MM: multiple myeloma; MyD88: myeloid differentiation primary response 88; NHL: non-Hodgkin’s lymphoma; NSCLC: non-small cell lung cancer; R/R: relapsed/refractory; SCLC: small cell lung cancer; SLL: small lymphocytic lymphoma.

Time	Degrader	Target	Indication	NCT Number	Phase
2011 2014 2015 2015 2015 2016 2017 2017 2017	Avadomide	IKZF1/3	NHL, DLBCL, hepatocellular carcinoma, B-cell CLL, MM, CLL/SLL, follicular lymphoma, advanced solid tumors and melanoma	NCT01421524 NCT02031419 NCT02417285 NCT02406742 NCT02509039 NCT02859324 NCT03310619 NCT03283202 NCT03834623	Phase I Phase I Phase I Phase I/II Phase I Phase I/II Phase I/II Phase I/II Phase II
2016 2020	CC-90009	GSPT1	R/R AML or R/R higher-risk myelodysplastic syndromes	NCT02848001 NCT04336982	Phase I Phase I
2017 2019 2022 2022 2022	CC-92480	IKZF1/3	R/R MM	NCT03374085 NCT03989414 NCT05372354 NCT05519085 NCT05552976	Phase I/II Phase I/II Phase I/II Phase III Phase III
2017 2019 2020 2021 2021	CC-99282	IKZF1/3	R/R NHL and CLL/SLL	NCT03310619 NCT03930953 NCT04434196 NCT04884035 NCT05169515	Phase I/II Phase I Phase I Phase I Phase I
2021	CFT7455	IKZF1/3	R/R NHL or MM	NCT04756726	Phase I/II
2019	DKY709	IKZF2/4	Advanced solid tumors	NCT03891953	Phase I
2016 2017 2020 2020 2020 2021 2021 2021 2021 2021 2021 2021 2022 2022 2022 2022 2022 2022 2022 2022 2022 2022	Iberdomide	IKZF1/3	Newly diagnosed MM, R/R MM, (smoldering) plasma cell myeloma and R/R lymphoma	NCT02773030 NCT03310619 NCT04392037 NCT04464798 NCT04564703 NCT04776395 NCT04855136 NCT04998786 NCT04884035 NCT04934475 NCT04975997 NCT05169515 NCT05177536 NCT05199311 NCT05272826 NCT05289492 NCT05392946 NCT05354557 NCT05434689 NCT05527340 NCT05558319 NCT05560399	Phase I/II Phase I/II Phase II Phase I/II Phase II Phase II Phase I/II Phase II Phase I Phase III Phase III Phase I Phase II Phase I/II Phase II Phase I/II Phase I/II Phase II Phase I/II Phase II Phase II Phase I
2022	KT-413	IRAK4	R/R B-cell NHL and MYD88 mutant and MYD88 wild-type R/R DLBCL	NCT05233033	Phase I
2022	MRT-2359	GSPT1	NSCLC, SCLC, high-grade neuroendocrine cancer of any primary site, DLBCL and tumors with L-MYC or N-MYC amplification	NCT05546268	Phase I/II

**Table 5:** Summary of degrader drugs under clinical evaluation in other disease entities. Red: active, not recruiting; yellow: recruiting; blue: completed; violet: not yet recruiting. AD: atopic dermatitis; AGA: androgenetic alopecia; AR: androgen receptor; HS: hidradenitis suppurativa; IKZF: IKAROS zinc-finger; IRAK4: interleukin-1 receptor-associated kinase 4; SLE: systemic lupus erythematosus.

Time	Degrader	Target	Indication	NCT Number	Phase
2017 2021	Avadomide	IKZF1/3	Renal insufficiency Critical illness and sepsis	NCT03097016 NCT05083520	Phase I
2022	GT20029	AR	Acne vulgaris and AGA	NCT05428449	Phase I
2014 2017 2019 2021 2021	Iberdomide	IKZF1/3	SLE SLE Hepatic impairment Renal insufficiency Critical illness and sepsis	NCT02185040 NCT03161483 NCT03824678 NCT04933747 NCT05083520	Phase II Phase II Phase I Phase I
2021	KT-474	IRAK4	AD and HS	NCT04772885	Phase I

## Abbreviations

2H2022	Second half of the year 2022
AbTAC	Antibody-based PROTAC
AD	Atopic dermatitis
ADMET	Absorption, distribution, metabolism, excretion and toxicity
AGA	Androgenetic alopecia
AhR	Aryl hydrocarbon receptor
AID	Auxin-inducible degron
ALK	Anaplastic lymphoma kinase
AML	Acute myeloid leukemia
AR	Androgen receptor
ASGPR	Asialoglycoprotein receptor
ASO	Antisense oligonucleotide
aTAG ATP	AchillesTag Adenosine triphosphate
ATTEC	Autophagy tethering compound
AUTAC	Autophagy targeting chimera
AUTOTAC	Autophagy targeting chimera
Bcl	B-cell lymphoma
BCL-xL	B-cell lymphoma-extra large
BCR	Breakpoint cluster region protein
BET	Bromo- and extra-terminal family
BIAC	Bispecific aptamer chimera
BRAF	V-raf murine sarcoma viral oncogene homolog B1
BRD	Bromodomain-containing protein
BTK	Bruton’s tyrosine kinase
CAR	Chimeric antigen receptor
CDC20	Cell division cycle 20
CDK	Cyclin-dependent kinase
Cer	Ceritinib
cIAP	Cellular inhibitor of apoptosis
CI-M6PR	Cation-independent mannose-6-phosphonate receptor
CK1α	Casein kinase 1α
CK2	Casein kinase 2
CLIPTAC	In-cell-click-formed proteolysis targeting chimera
CLL	Chronic lymphocytic leukemia
CMA	Chaperone-mediated autophagy
CPP-LSS	Cell-penetrating peptide and lysosome-sorting sequence
CRABP I/II	Cellular retinoic acid-binding protein 1/2
CRBN	Cereblon
CREPT	Cell cycle-related and expression elevated protein in tumor
CRISPR	Clustered regularly interspaced short palindromic repeats
CT	Computed tomography
CYP1B1 DCAF5	Cytochrome p450 family 1 subfamily B member 1 DDB1 and CUL4 associated factor 5
DCAF	DDB1 and CUL4 associated factor
DHODH	Dihydroorotate dehydrogenase
DHX36	DEAH-box-protein 36
DLBCL DNA	Diffuse large B cell lymphoma Deoxyribonucleid acid
dsDNA	Double-stranded DNA
dTAG	Degrader tag
DUB	Deubiquitinating enzyme
DUBTAC	Deubiquitinase-targeting chimera
eEF2K	Eukaryotic elongation factor 2 kinase
EGFR	Epidermal growth factor receptor
eIF4E	Eukaryotic initiating factor 4E
EML4	Echinoderm microtubule-associated protein-like 4
ER	Estrogen receptor
ErbB3	Erb-b2 receptor tyrosine kinase 3
ERK	Extracellular signal-regulated kinase
ESR1	Estrogen receptor 1
EZH2	Enhancer of zeste homolog 2
FAK	Focal adhesion kinase
FDA	Food and drug administration
FEM1B	Feminization-1 homolog b
FKBP	FK506-binding proteins
FLT3	Fms-like tyrosine kinase 3
FOXO1 GlueTAC	Forkhead box protein O1 GlueBody Chimera
GNPs	Gold nanoparticles
GPD1L	Glycerol-3-phosphate dehydrogenase 1 like protein
GREB1	Growth regulating estrogen receptor binding 1
GSTP1 G4	G_1_ to S phase transition 1 G-quadruplex
HDAC	Histone deacetylase
HECT	Homology to E6AP C terminus
HER	Human epidermal growth factor receptor
HIF-1-alpha	Hypoxia-inducible factor 1-alpha
HIP1R	Huntingtin-interacting protein 1–related
HPK1	Hematopoietic progenitor kinase 1
HS HyT IAP	Hidradenitis suppurativa Hydrophobic tagging Inhibitors of apoptosis protein
IDO1	Indoleamine 2,3-dioxygenase 1
IGF-1R	Insulin-like growth factor 1 receptor
IKZF	IKAROS zinc finger family
IMiD	Immunomodulatory drug
IND	Investigational new drug
IRAK4	Interleukin-1 receptor-associated kinase 4
ITK IV	Interleukin-2-inducible T-cell kinase Intravenous injection
JAK	Janus kinase
JAMM	Jab1/MPN domain-associated metallopeptidase
KEAP1	Kelch-like ECH associated protein 1
LC3	Microtubule-associated protein 1A/1B-light chain 3
LD	Lipid droplets
LEF1	Lymphoid enhancer binding factor 1
LGL	Large granular lymphocyte
LHRH	Luteinizing hormone-releasing hormone
LIR	LC3-interacting region
LTR	Lysosomal targeted receptor
LYTAC	Lysosome targeting chimera
MCL	Mantle cell lymphoma
Mcl-1	Myeloid cell leukemia-1
MCPIP	Monocyte chemotactic protein-induced protein
mCRPC	Metastatic castration-resistant prostate cancer
MDM2	Mouse double minute 2
MEK	Mitogen-activated protein kinase kinase
Met	Mesenchymal epithelial transition
mHTT	Mutant huntingtin
MINDY	Motif interacting with Ub-containing novel DUB family
miR-96	MicroRNA 96
MJD	Machado–Joseph disease protein domain
MM	Multiple myeloma
MoDE-A	Molecular degrader of extracellular proteins through the ASGPR
MRI	Magnetic resonance imaging
MTH1	MutT homolog-1
MyD88	Myeloid differentiation primary response 88
NF-κB	Nuclear factor kappa-light-chain-enhancer of activated B cells
NHL	Non-Hodgkin’s lymphoma
NRAS	Neuroblastoma RAS viral oncogene homolog
NSCLC	Non-small cell lung cancer
NTRK	Neurotrophic tyrosine receptor kinase
NVOC	Nitroveratryloxycarbonyl
OTU	Ovarian tumor protease
PARP1	Poly [ADP-ribose] polymerase 1
PB1	Phox and Bem1 domain
PDE	Phosphodiesterase
PD-L1	Programmed death-ligand 1
PDX	Patient derived xenograft
PEG	Polyethylene glycol
PHOTAC	Photochemically targeting chimera
PI3K	Phosphoinositide 3-kinases
PLK1 PO	Polo-like kinase 1 Per os
POI	Protein of interest
POLY-PROTAC	Polymeric PROTAC
Pom	Pomalidomide
PR	Progesterone receptor
PRC	Polycomb repressive complex
PRMT5	Protein arginine methyltransferase 5
PROTACs	Proteolysis targeting chimeras
PTB	Phosphotyrosine-binding
PTK-7	Protein kinase-like 7
R/R	Relapsed/refractory
RAS	Rat sarcoma
RBFOX1	RNA binding Fox-1 homolog 1
RBM23	RNA binding motif protein 23
RBM39	RNA binding motif protein 39
RBP	RNA binding protein
RBR	RING-between-RING
RHAU	RNA helicase associated with AU-rich element
RIBOTAC	Ribonuclease Targeting chimera
RING	Really interesting new gene
RIPK2 RNA	Receptor interacting serine/threonine kinase 2 Ribonucleic acid
RNF	RING finger protein
RTK SC	Receptor tyrosine kinase Subcutaneous injection
SCLC	Small cell lung cancer
SERD	Selective estrogen receptor degrader
SGK	Serum/glucocorticoid regulated kinase family member
SH2	Src homology 2
SHP2	Src homology 2 domain containing protein tyrosine phosphatase-2
siRNA	Small interfering RNA
SirT2	Sirtuin 2
SLC9A1	Solute carrier family 9 member A1
SLE	Systemic lupus erythematosus
SLL	Small lymphocytic lymphoma
SMARCB1	SWI/SNF related, matrix associated, actin dependent regulator of chromatin, subfamily B, member 1
SNIPER	Specific and non-genetic inhibitors of apoptosis protein (IAP)-dependent protein eraser
SPAAC	Strain-promoted azide–alkyne cycloaddition
SRC-1	Steroid receptor coactivator-1
STAT3	Signal transducer and activator of transcription 3
TACC3	Transforming acidic coiled-coil containing protein 3
TBK1	TANK-binding kinase 1
TCF	T-cell factor
TCL	T-cell lymphomas
TCO	Trans-cyclooctene
TF	Transcription factor
TFF	Trefoil factor
TGF-β1	Transforming growth factor beta 1
TI	Tumor-infiltrating
TOI	Transcription factor of interest
TPD	Targeted protein degradation
TPS	Targeted protein stabilization
TRAFTAC	Transcription factor targeting Chimera
Treg	Regulatory T cell
TRIM24	Tripartite motif containing 24
TrkA	Tropomyosin receptor kinase A
TZ	Tetrazine
Ub	Ubiquitin
UCH	Ubiquitin C-terminal hydrolase
UPS	Ubiquitin-proteasome system
USP	Ubiquitin-specific protease
UV	Ultraviolet
VEGFR	Vascular endothelial growth factor receptor
VHL	Von Hippel-Lindau
WM	Waldenström macroglobulinaemia
XIAP	X-linked inhibitor of apoptosis protein
ZUFSP	Zn-finger and UFSP domain protein
α1A-AR	Alpha-1A adrenergic receptor
